# Supplementary material for: Serotonergic modulation of vigilance states in zebrafish and mice
Source: Nat Commun. 2024 Mar 22;15:2596. doi: 10.1038/s41467-024-47021-0 (PMC10959952; doi:10.1038/s41467-024-47021-0)
Supplement: Supplementary file 1 — Supplementary information [file 41467_2024_47021_MOESM1_ESM.pdf]

Serotonergic modulation of vigilance states in zebrafish and mice

Supplementary information

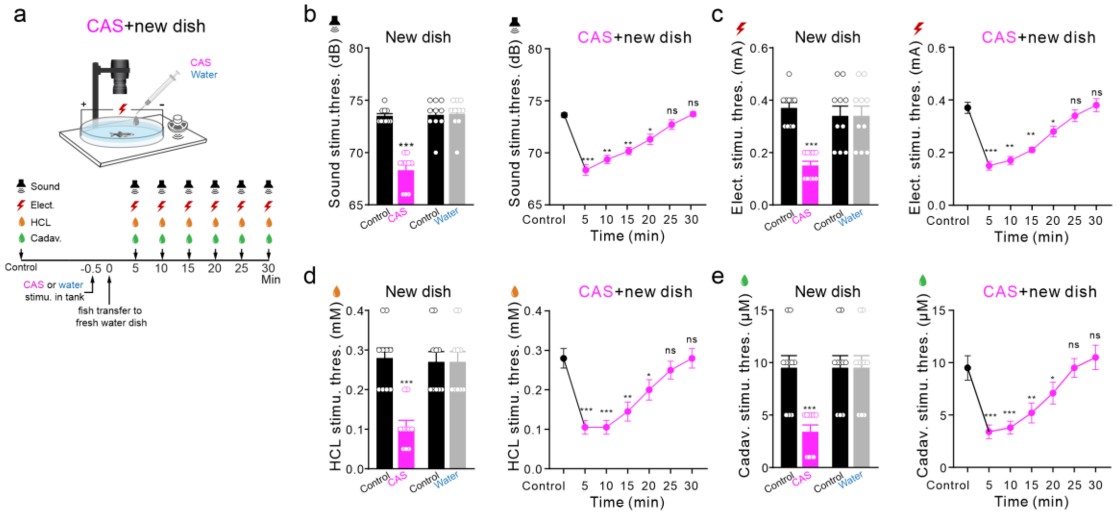

Supplementary Fig. 1. CAS-induced vigilance behavior persisted after transfer to the new dish. a Illustration showing timeline for stimulating and then transferring the stimulated fish into a new fresh water dish. Note that the CAS-induced reduction in response thresholds persisted after transfer to the new dish where there was no CAS present. **b-e** Left panels compare the response thresholds to aversive stimuli before and 5 min after transferring CAS/water-treated fish into the fresh water dish. Right panels show mean data of the prolonged change of response thresholds to each stimulus after transferring into the fresh water dish after CAS treatment for 30 min. N= 10 Fish in each group. All data are presented as mean ± SEM. **P* < 0.05, ***P* < 0.01, ****P* < 0.001; ns denoting no significant difference. For detailed statistics, see Supplementary Table 2. Source data are provided as a Source Data file.

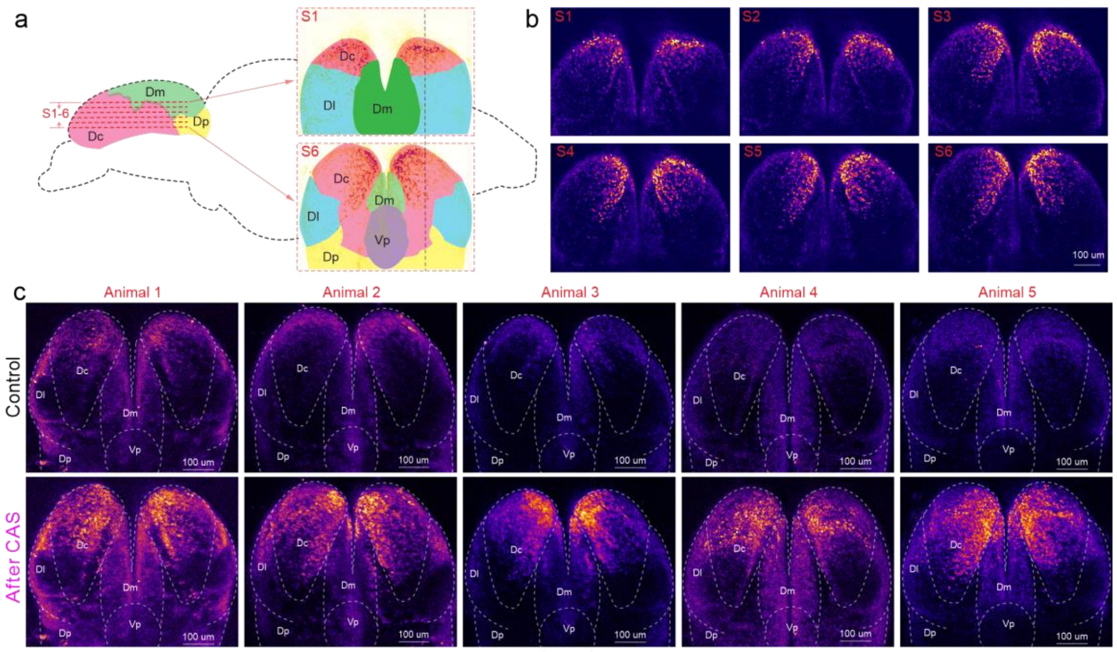

Supplementary Fig. 2. Two-photon images of 6-layer volume scan of zebrafish pallial Dc region after CAS treatment. a Drawing describing how the 6-layer volume scan of zebrafish dorsal pallium was obtained and brain regions in layer 1 and 6. **b** Two-photon images of 6 distinct layers in zebrafish dorsal pallium showing spatiotemporal calcium pattern of synchronized neuronal activities in Dc region after CAS treatment. **c** Combined stacked calcium images of the 6 layers in dorsal pallium before and after CAS treatment are shown for five fish.

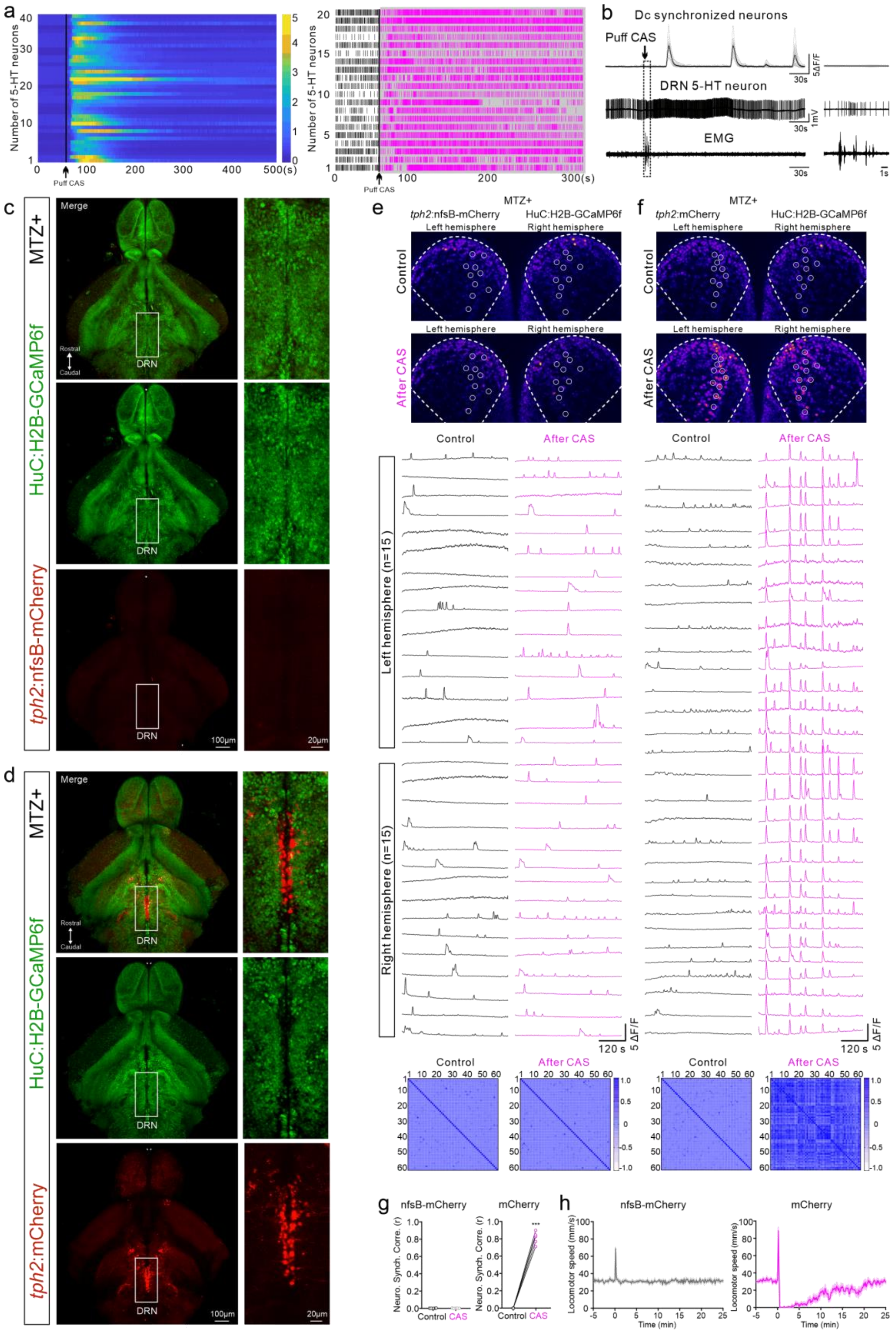

Supplementary Fig. 3. Chemogenetic ablation of 5-HT neurons abolished CAS-induced neuronal synchrony and vigilance behavior. a Heatmap of calcium dynamics (left, related to Fig. 3b-c) and raster plot of action potentials recorded in 5-

HT neurons (right, related to Fig. 3h) after CAS treatment. **b** The experiments demonstrated that the erratic swim (EMG) occurred first, then the intense activation of DRN 5-HT neurons followed and finally the neuronal synchrony in Dc appeared. **c-d** Immunofluorescence images showing ablation of 5-HT neurons after MTZ (5 mM) treatment for five days in *tph2:nfsB-mCherry* crossed with *HuC:H2B-GCaMP6f* fish (b). *tph2:mCherry* crossed with *HuC:H2B-GCaMP6f* fish was used as a negative control, showing no changes after MTZ treatment (c). **e-f** Two-photon calcium images and the derived data show that CAS administration failed to generate synchronized neuronal oscillation in Dc region of *tph2:nfsB-mCherry* crossed with *HuC:H2B-GCaMP6f* fish after MTZ treatment (d). The CAS-induced neuronal synchronization normally observed in Dc region of *tph2:mCherry* crossed with *HuC:H2B-GCaMP6f* fish (e). **g** Statistical analysis demonstrating the correlation coefficient in e-f. Each point represents the mean value of one fish. N=5 fish. **h** Quantification of locomotor speed in 10 fish treated with MTZ after CAS administration for 30 minutes for the in *tph2:nfsB-mCherry* fish and *tph2:mCherry* fish as a negative control. All data are presented as mean ± SEM. $^{***}P < 0.001$. For detailed statistics, see Supplementary Table 2. Source data are provided as a Source Data file.

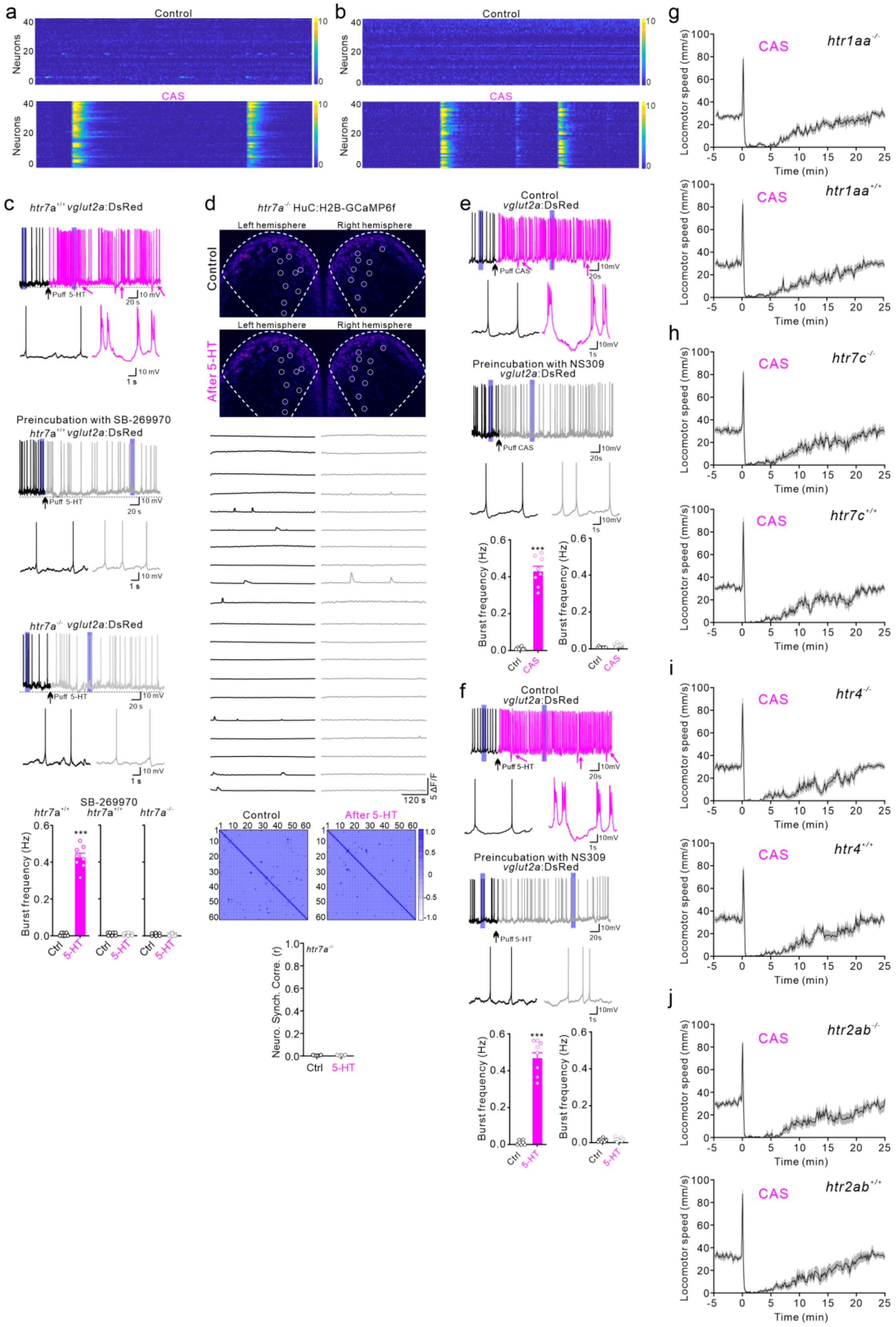

Supplementary Fig. 4. The released 5-HT generated internal synchronized state through acting on 5-HTR7 of glutamatergic neurons in Dc region. a Heat maps of calcium dynamics for Fig. 4c. **b** Heat maps of calcium dynamics for Fig.4d. **c** Puffing

5-HT induced bursting firing and post-inhibitory rebound in glutamatergic neurons in Dc region of *htr7a*$^{+/+}$ *vglut2a:DsRed* fish (upper pair of panels). Preincubation of SB-269970 (0.3 μM, middle pair of panels) or using *htr7*$^{-/-}$ *vglut2a:DsRed* fish (lower pair of panels) prevented bursting firing and post-inhibitory rebound in glutamatergic neurons in Dc region puffed with 5-HT. Blue highlighted sections of traces are enlarged and shown underneath. N= 8 fish in each group. **d** In *htr7a*$^{-/-}$ *HuC:H2B-GCaMP6f* fish puffing 5-HT on neurons in Dc region failed to generate synchronized neuronal oscillations. Two-photon calcium imaging was carried on the circled neurons and the analyzed data of the individual cells are similar in the absence or presence of 5-HT. **e-f** Preincubation of NS 309 (a SK channel activator, 100 μM) inhibited CAS-induced (panel e) or 5-HT-induced (panel f) bursting firing and post-inhibitory rebound in glutamatergic neurons in Dc region of *vglut2a:DsRed* fish. Shadow highlighted sections of traces are expanded and shown underneath. N= 8 fish in each group. **g-j** Mean data of locomotor speed of *htr1aa*$^{-/-}$, *htr7c*$^{-/-}$, *htr4*$^{-/-}$, *htr2ab*$^{-/-}$and their sibling groups in response to CAS stimulation. N= 10 fish in each group. All data are presented as mean ± SEM. ***$P < 0.001$. For detailed statistics, see Supplementary Table 2. Source data are provided as a Source Data file.

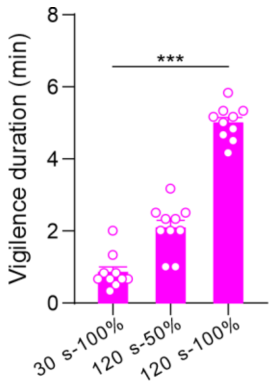

Supplementary Fig. 5. Optogenetic activation of DRN 5-HT neurons induces vigilance behavior in a time and intensity dependent manner.

Mean duration of vigilance behavior state induced by different blue light stimulation protocols. Each circle represents one fish. N = 10 fish in each group. 100% light intensity equals 15mW LED power. All data are presented as mean ± SEM. ***$P < 0.001$, significant difference. For detailed statistics, see Supplementary Table 2. Source data are provided as a Source Data file.

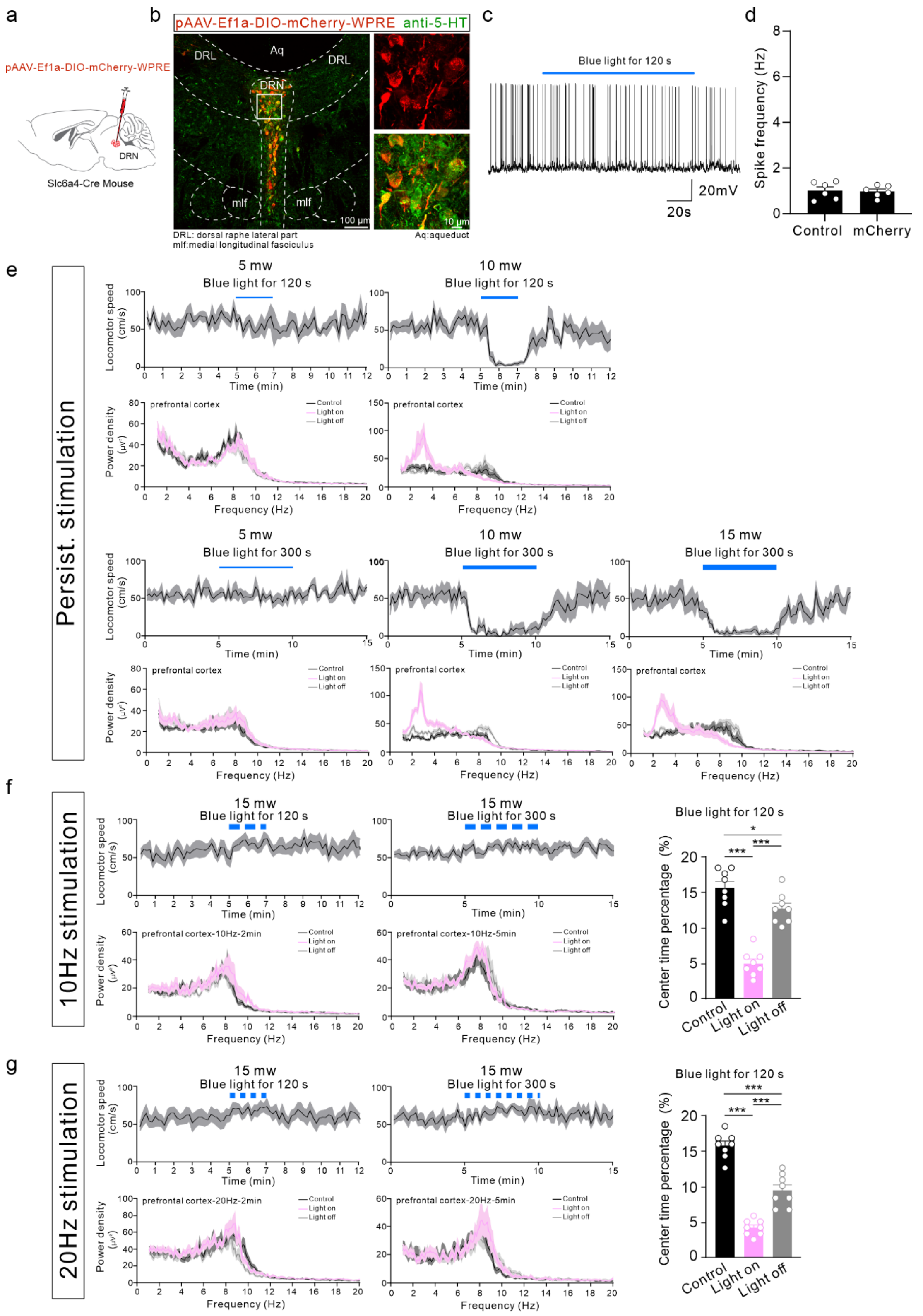

Supplementary Fig. 6. Persistent optogenetic activation of DRN 5-HT neurons induce internal synchronized state and vigilance behavior in mice. a Illustration of stereotaxic injection of the Cre-dependent AAV control virus into the DRN of Slc6a4-Cre mice. **b** Immunofluorescence image showing co-localization of mCherry and anti-

5-HT staining in mice DRN. White banded area is enlarged to show separately mCherry and anti-5-HT staining. **c-d** Blue light stimulation had no effect on the recorded activity in DRN 5-HT neurons in the control virus group. n=6 neurons from 3 mice for each group. **e** Quantification of locomotor speed and EEG power spectra density analysis in response to blue light stimulation protocols with different duration or intensity in the Slc6a4-hChR2 mice. **f-g** (left) Quantification of locomotor speed and EEG power spectra density analysis in response to 10 Hz 15 mW (f) or 20 Hz 15 mW(g) pulse stimulation for 120 s or 300 s in the Slc6a4-hChR2 mice. (right) The mean percentage time spent by mice in the central region of the open field before, during and after pulse stimulation in the Slc6a4-hChR2 mice. N=8 mice in each group. All data are presented as mean ± SEM. $*P < 0.05$, $***P < 0.001$. For detailed statistics, see Supplementary Table 2. Source data are provided as a Source Data file.

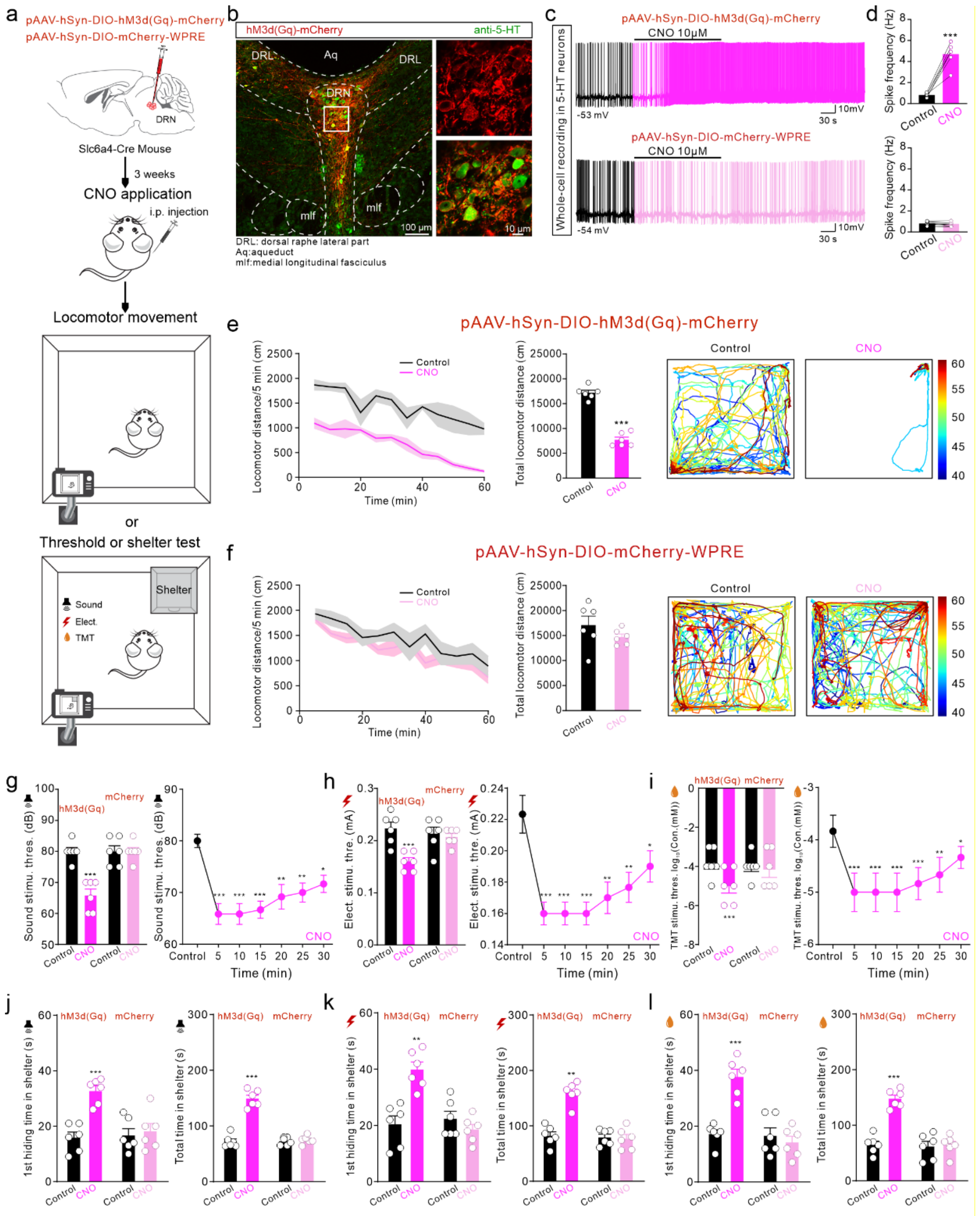

Supplementary Fig. 7. Chemogenetic activation of DRN 5-HT neurons generated and maintained vigilance behavior in mice. a Drawing showing stereotaxic injection of the Cre-dependent AAV virus in DRN, chemogenetic activation of DRN 5-HT neurons by CNO injection and behavior tests in mice in a timeline. **b** Immunofluorescence image showing expression of hM3d(Gq)-mCherry in DRN 5-HT neurons. White banded area is enlarged to show separately mCherry and anti-5-HT staining. **c-d** Spike discharge was increased by CNO in Slc6a4-hM3d(Gq) but not Slc6a4-mCherry mice DRN 5-HT neurons. Representative traces (c) and mean spike

frequency (d) are shown before and after CNO administration in. n=6 neurons from 3 mice in each group. **e-f** Distance travelled after CNO administration was greatly reduced in Slc6a4-hM3d(Gq) (e) but unaffected in Slc6a4-mCherry (f) mice. Mean locomotor distance in 1-hour recording before and after CNO administration (middle panels). Representative control and post CNO locomotor tracks in the last 20 minutes of 1-hour recording for one mouse in each group is shown in the extreme right panels. The color bar indicates recording time. **g-i** Mean data showing reduced thresholds to sound (g), electric shock (h), and TMT stimulation (i) after chemogenetic activation (from 40 min after CNO injection) of DRN 5-HT neurons in Slc6a4-hM3d(Gq) mice but not Slc6a4-mCherry mice (left panel). Mean data illustrating the persistent nature (for at least 30 min) of the reduced response thresholds to aversive stimuli after chemogenetic activation is shown in the right panel. N = 6 mice for each group. **j-l** Mean stay time in the shelter from the first entry (left) or the entire process (right) in response to sound (j), electric shock (k) and TMT (l) after mice received CNO was increased in Slc6a4-hM3d(Gq) but not in Slc6a4-mCherry mice. N = 6 mice in each group. All data are presented as mean ± SEM. $*P < 0.05$, $**P < 0.01$, $***P < 0.001$. For detailed statistics, see Supplementary Table 2. Source data are provided as a Source Data file.

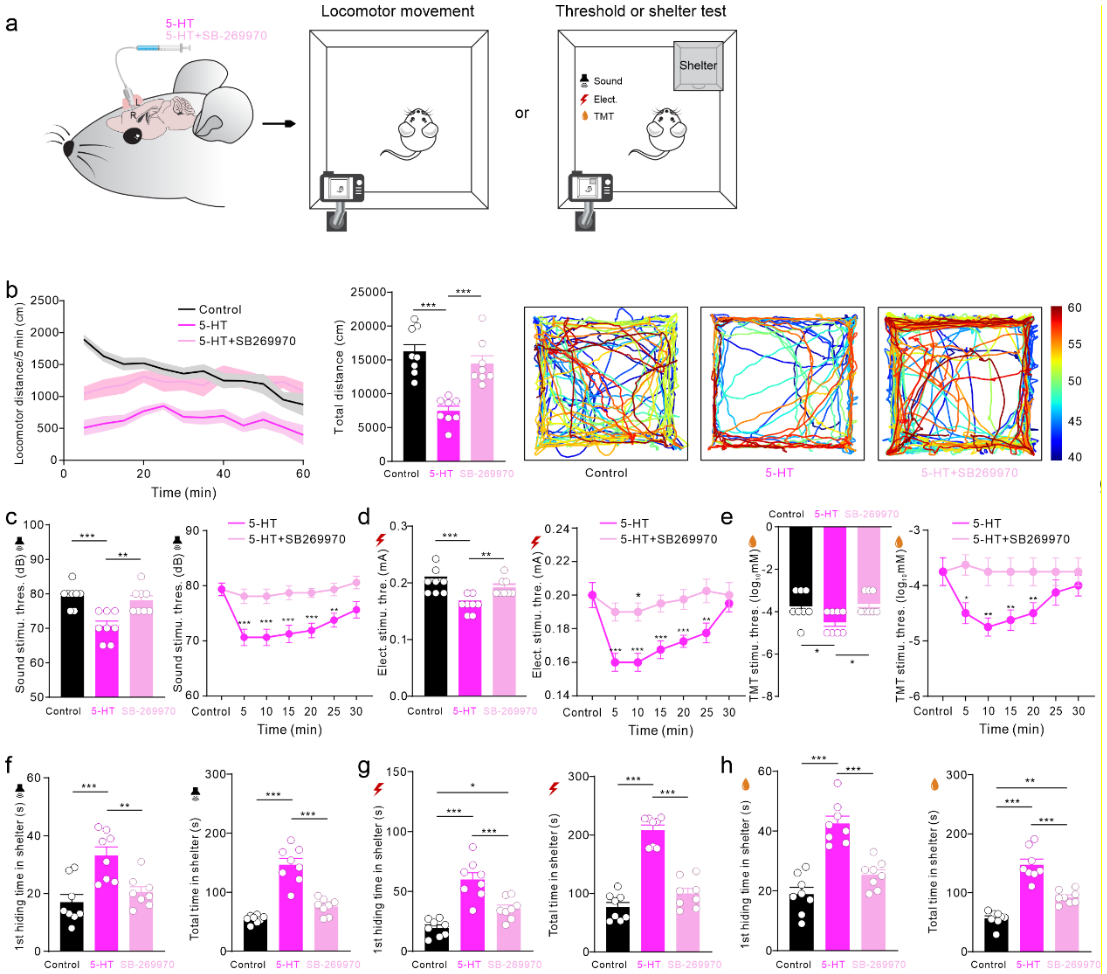

Supplementary Fig. 8. Exogenous administration of 5-HT generated and maintained vigilance behavior in mice. **a** Illustration of administration of 5-HT by an intracerebral inserted cannula into the prefrontal cortex and the movement and shelter behavior tests in mice. **b** Distance travelled before and after administration of 5-HT or 5-HT together with SB269970 (left panel). Average locomotor distance during one hour before and after drug administration (middle panel). Typical locomotor tracking of one animal in each group during the last 20 minutes of recording (right panel). N = 8 mice for each group. **c-e** Mean response thresholds to sound (c), electric shock (d), and TMT stimuli (e) before and after administration of 5-HT or 5-HT together with SB269970 (left panel). Average data showing the enduring change in response thresholds to aversive stimuli after drug administration (right panel). N = 6 mice for each group. **f-h** Mean stay time in the shelter from the first entry (left panel) or the entire process (right panel) in response to sound (f), electric shock (g) and TMT (h) before and after administration of 5-HT or 5-HT mixed with SB269970. N = 6 mice in each group. All

data are presented as mean ± SEM. *$P < 0.05$, **$P < 0.01$, ***$P < 0.001$. For detailed statistics, see Supplementary Table 2. Source data are provided as a Source Data file.

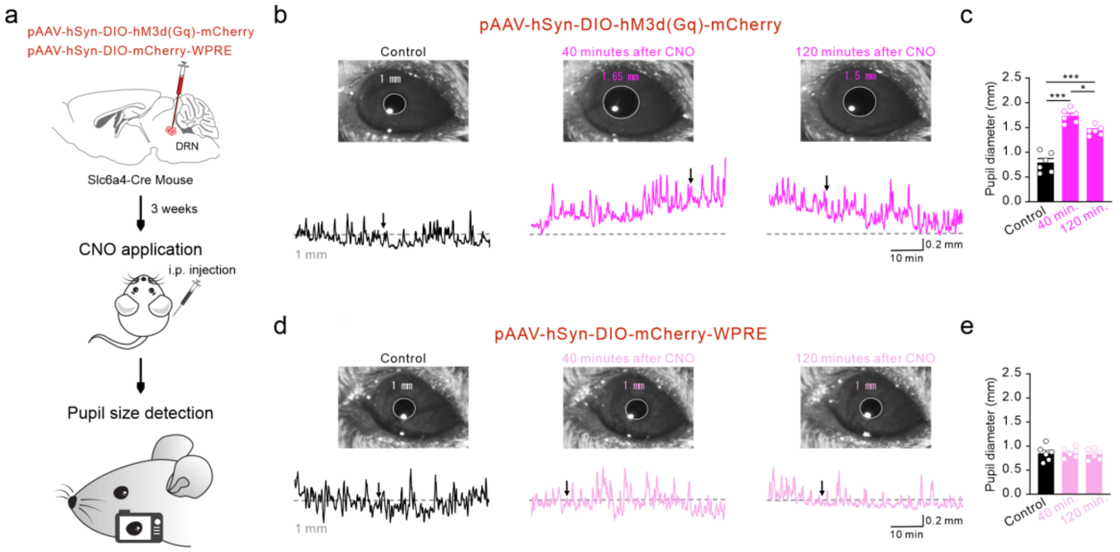

Supplementary Fig. 9. Chemogenetic activation of DRN 5-HT neurons persistently increased pupillary size in mice. a Illustration of pupillometry and chemogenetic activation of DRN 5-HT neurons. **b-c** Representative pupil images (indicated by arrow) and pupillary size fluctuations in Slc6a4-hM3d(Gq) mice before, 40 min and 120 min after CNO application (b). Each recording lasted for 60 min. Mean pupil size was increased in Slc6a4-hM3d(Gq) mice for up to 120 min after CNO application (c). **d-e** Representative pupil images and pupillary size fluctuations in Slc6a4-mCherry mice before, 40 min and 120 min after CNO application (d). Each recording lasted for 60 min. Mean pupil size was unchanged in Slc6a4-mCherry mice after CNO application (e). All data presented as mean ± SEM. $*P < 0.05$, $***P < 0.001$. For detailed statistics, see Supplementary Table 2. Source data are provided as a Source Data file.

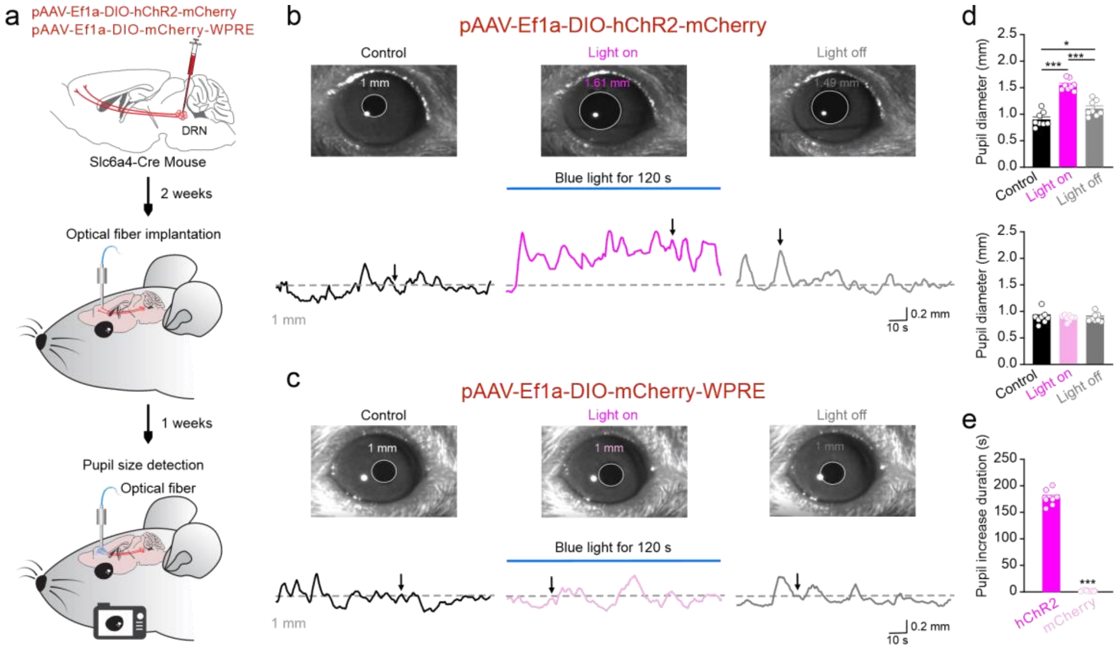

Supplementary Fig. 10. Persistent optogenetic activation of DRN 5-HT neuron terminals in PFC increased pupillary size in mice. a Illustration of pupillometry and optogenetic stimulation of DRN 5-HT neuron terminals in PFC. **b-c** Representative pupil images (indicated by arrow) and pupillary size fluctuations for 120 s in Slc6a4-hChR2 (b) and Slc6a4-mCherry (c) mice before, during and after optogenetic activation of DRN 5-HT neuron terminals in PFC. **d** Mean data showing an increased pupillary size after optogenetic stimulation in Slc6a4-hChR2 but not Slc6a4-mCherry mice. **e** Mean data showing the duration of pupillary increase after optogenetic stimulation in Slc6a4-hChR2 and Slc6a4-mCherry mice. N = 8 mice in each group. All data are presented as mean ± SEM. $**P < 0.01$, $***P < 0.001$. For detailed statistics, see Supplementary Table 2. Source data are provided as a Source Data file.

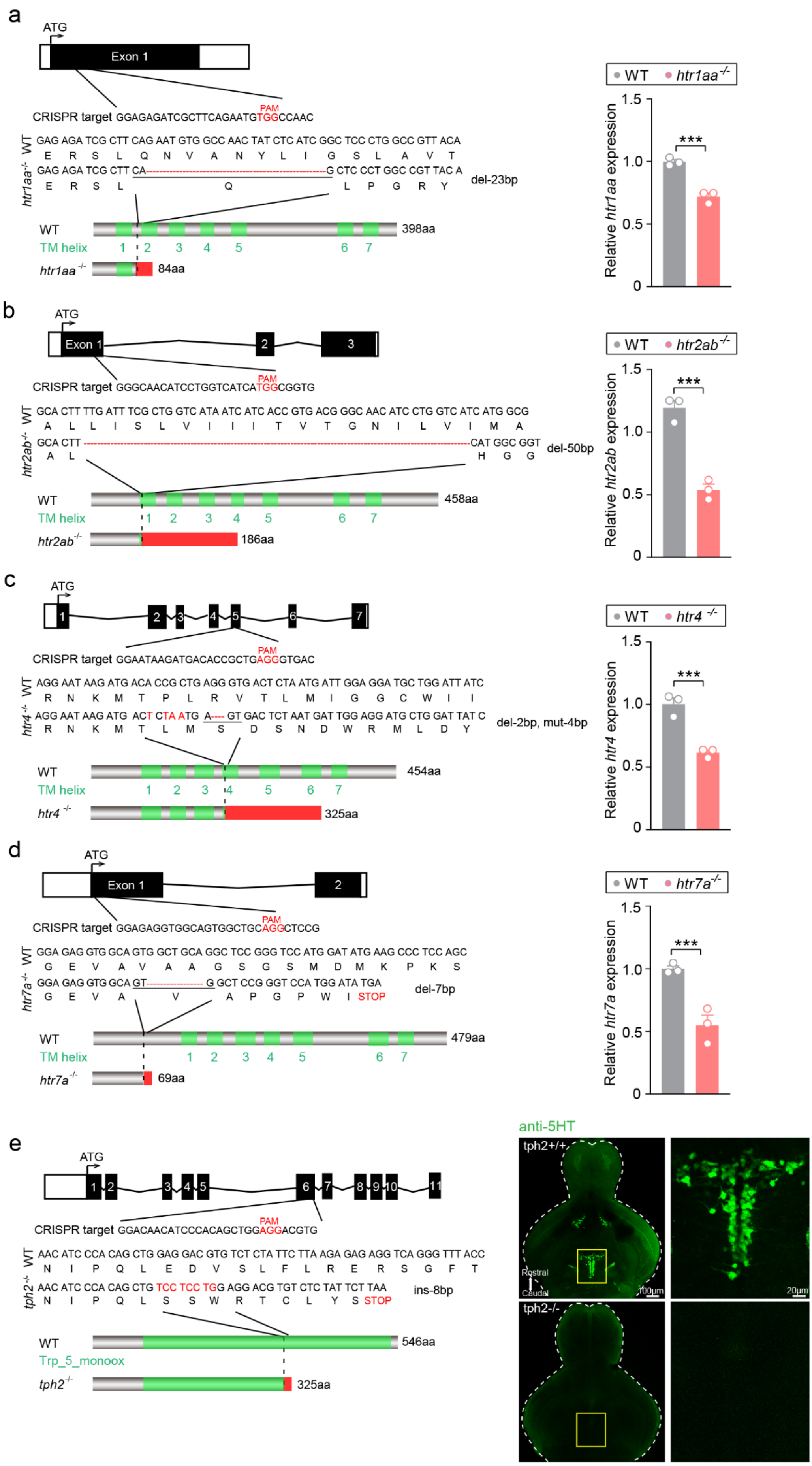

a
ATG
Exon 1
PAM
CRISPR target GGAGAGATCGCTTCAGAATGTGGCCAAC
WT
GAG AGA TCG CTT CAG AAT GTG GCC AAC TAT CTC ATC GGC TCC CTG GCC GTT ACA
E R S L Q N V A N Y L I G S L A V T
htr1aa-/-
GAG AGA TCG CTT CA------G CTC CCT GGC CGT TAC A
E R S L Q L P G R Y
del-23bp
WT
TM helix
1 2 3 4 5 6 7
398aa
htr1aa-/-
84aa
WT htr1aa-/-
Relative htr1aa expression
1.5
1.0
0.5
0
***
b
ATG
Exon 1
2
3
PAM
CRISPR target GGGCAACATCCTGGTCATCATGGCGGTG
WT
GCA CTT TTG ATT TCG CTG GTC ATA ATC ATC ACC GTG ACG GGC AAC ATC CTG GTC ATC ATG GCG
A L L I S L V I I I T V T G N I L V I M A
htr2ab-/-
GCA CTT ------CAT GGC GGT
A L H G G
del-50bp
WT
TM helix
1 2 3 4 5 6 7
458aa
htr2ab-/-
186aa
WT htr2ab-/-
Relative htr2ab expression
1.5
1.0
0.5
0
***
c
ATG
1 2 3 4 5 6 7
PAM
CRISPR target GGAATAAGATGACACCGCTGAGGGTGAC
WT
AGG AAT AAG ATG ACA CCG CTG AGG GTG ACT CTA ATG ATT GGA GGA TGC TGG ATT ATC
R N K M T P L R V T L M I G G C W I I
htr4-/-
AGG AAT AAG ATG ACT CTA ATG A---- GT GAC TCT AAT GAT TGG AGG ATG CTG GAT TAT C
R N K M T L M S D S N D W R M L D Y
del-2bp, mut-4bp
WT
TM helix
1 2 3 4 5 6 7
454aa
htr4-/-
325aa
WT htr4-/-
Relative htr4 expression
1.5
1.0
0.5
0
***
d
ATG
Exon 1
2
PAM
CRISPR target GGAGAGGTGGCAGTGGCTGCAGGCTCCG
WT
GGA GAG GTG GCA GTG GCT GCA GGC TCC GGG TCC ATG GAT ATG AAG CCC TCC AGC
G E V A V A A G S G S M D M K P K S
htr7a-/-
GGA GAG GTG GCA GT------G GCT CCG GGT CCA TGG ATA TGA
G E V A V A P G P W I STOP
del-7bp
WT
TM helix
1 2 3 4 5 6 7
479aa
htr7a-/-
69aa
WT htr7a-/-
Relative htr7a expression
1.5
1.0
0.5
0
***
e
ATG
1 2 3 4 5 6 7 8 9 10 11
PAM
CRISPR target GGACAACATCCCACAGCTGGAGGACGTG
WT
AAC ATC CCA CAG CTG GAG GAC GTG TCT CTA TTC TTA AGA GAG AGG TCA GGG TTT ACC
N I P Q L E D V S L F L R E R S G F T
tph2-/-
AAC ATC CCA CAG CTG TCC TCC TGG AGG ACG TGT CTC TAT TCT TAA
N I P Q L S S W R T C L Y S STOP
ins-8bp
WT
Trp_5_monoox
546aa
tph2-/-
325aa
anti-5HT
tph2+/+
Rostral
Caudal
100μm
20μm
tph2-/-

Supplementary Fig. 11. Genetic knock-out of 5-HT receptor coding genes in zebrafish. a Left panel shows the Htr1aa protein containing a 7tm_GPCRs domain (398 aa), and the truncated short peptide of *htr1aa* mutant (84 aa). Mutation and indel are indicated in red. Right panel shows decreased *htr1aa* expression in *htr1aa*$^{-/-}$ animals compared to wild-type siblings. Relative expression was normalized to *gapdh* levels. n= 3 technical replicates of 20 pooled fish, the experiments was performed in triplicate. **b** Generation of *htr2ab* mutant (left) and qRT-PCR detection of relative expression of *htr2ab* (right). **c** Generation of *htr4* mutant (left) and qRT-PCR detection of relative expression of *htr4* (right). **d** Generation of *htr7a* mutant (left) and qRT-PCR detection of relative expression of *htr7a* (right). **e** Generation of *tph2* mutant (left) and immunostaining detection of anti-5-HT in mutant or sibling (right). All data presented as mean ± SEM. $***P < 0.001$. For detailed statistics, see Supplementary Table 2. Source data are provided as a Source Data file.

Supplementary Table 1. All primers used in this study.

**Usage**	**Name**	**Sequence (5' - 3')**	**Source**
CRISPR gRNA primer	htr1aa-sg-F	GTAATACGACTCACTATAGGAGAGATCGCTTCAGAATGGTTTTAGAGCTAGAAATAGC	Sangon Biotech/This paper
genotyping	htr1aa-seq-F	AGCGAAGTTGCTCTGAGTTACC	Sangon Biotech/This paper
genotyping	htr1aa-seq-R	TGAAAATATCGCACATCTCCTG	Sangon Biotech/This paper
CRISPR gRNA primer	htr2ab-sg-F	GTAATACGACTCACTATAGGGCAACATCCTGGTCATCAGTTTTAGAGCTAGAAATAGC	Sangon Biotech/This paper
genotyping	htr2ab-seq-F	AGATTTCCAAAGACACCAGCAT	Sangon Biotech/This paper
genotyping	htr2ab-seq-R	TCCCAAAAGCATGTCTGTAATG	Sangon Biotech/This paper
CRISPR gRNA primer	htr4-sg-F	GTAATACGACTCACTATAGGAATAAGATGACACCGCTGGTTTTAGAGCTAGAAATAGC	Sangon Biotech/This paper
genotyping	htr4-seq-F	GCCTTTTCTTACAGGTACTATGCC	Sangon Biotech/This paper
genotyping	htr4-seq-R	CTGATTGCAAGGTGAGAAAGAA	Sangon Biotech/This paper
CRISPR gRNA primer	htr7a-sg-F	GTAATACGACTCACTATAGGAGAGGTGGCAGTGGCTGCGTTTTAGAGCTAGAAATAGC	Sangon Biotech/This paper
genotyping	htr7a-seq-F	AAAGCCACGGAGAAACTATGAA	Sangon Biotech/This paper
genotyping	htr7a-seq-R	CCTCTCCATAGCTGAGGATCTG	Sangon Biotech/This paper
CRISPR gRNA primer	tph2-sg-F	GTAATACGACTCACTATAGGACAACATCCCACAGCTGGGTTTTAGAGCTAGAAATAGC	Sangon Biotech/This paper
genotyping	tph2-seq-F	CGGTGGGTGCTTGTAGCTCAAT	Sangon Biotech/This paper
genotyping	tph2-seq-R	TAGTAATTCTGTTGAGAGACCGTGC	Sangon Biotech/This paper
CRISPR gRNA primer	sg-Common-R	AAAAGCACCGACTCGGTGCC	Sangon Biotech/This paper
qRT-PCR	qRT-htr1aa-F	AACAACACCACAGAAAGCCA	Sangon Biotech/This paper
qRT-PCR	qRT-htr1aa-R	ACCAGCACCGACACCATAA	Sangon Biotech/This paper
qRT-PCR	qRT-htr2ab-F	CCTCTACGGTTACACCTGGC	Sangon Biotech/This paper
qRT-PCR	qRT-htr2ab-R	GCTGTGGTGGATGGGGTT	Sangon Biotech/This paper
qRT-PCR	qRT-htr4-F	GTTGACGTGCTCAGTAGTGG	Sangon Biotech/This paper
qRT-PCR	qRT-htr4-R	AGTCTTGGCTGCCTTGGT	Sangon Biotech/This paper
qRT-PCR	qRT-htr7a-F	CACTCTTGGGATAGTAGTTGGA	Sangon Biotech/This paper
qRT-PCR	qRT-htr7a-R	TGAGGAGGCTTCGGTAGGT	Sangon Biotech/This paper

Supplementary Table 2. Detailed statistics.

Figure	Statistics	Result	Comparison	Significance	*P* value
**Fig 1e sound stimu.**	paired *t*-test (Two-tailed)	t=18.75, df=9	Control vs. CAS	***	*P* < 0.0001
	Descriptive	Control: 74.80 ± 0.4422 (n = 10 fish)			
		CAS: 67.70 ± 0.5783 (n = 10 fish)			
	paired *t*-test (Two-tailed)	t=0.8955, df=9	Control vs. Water	ns	*P* = 0.3938
	Descriptive	Control: 73.80 ± 0.4422 (n = 10 fish)			
		Water: 74.10 ± 0.4069 (n = 10 fish)			
**Fig 1e Elect. stimu.**	paired *t*-test (Two-tailed)	t=0.6530, df=9	Control vs. CAS	***	*P* = 0.0001
	Descriptive	Control: 0.4 ± 0.03944 (n = 10 fish)			
		CAS: 0.23 ± 0.02603 (n = 10 fish)			
	paired *t*-test (Two-tailed)	t=1.500, df=9	Control vs. Water	ns	*P* = 0.1679
	Descriptive	Control: 0.39 ± 0.03145 (n = 10 fish)			
		Water: 0.41 ± 0.0348 (n = 10 fish)			
**Fig 1e HCL stimu.**	paired *t*-test (Two-tailed)	t=9.391, df=9	Control vs. CAS	***	*P* < 0.0001
	Descriptive	Control: 0.28 ± 0.02906 (n = 10 fish)			
		CAS: 0.1050 ± 0.01740 (n = 10 fish)			
	paired *t*-test (Two-tailed)	t=0, df=9	Control vs. Water	ns	*P* > 0.9999
	Descriptive	Control: 0.29 ± 0.02333 (n = 10 fish)			
		Water: 0.29 ± 0.02333 (n = 10 fish)			
**Fig 1e Cadav. stimu.**	paired *t*-test (Two-tailed)	t=8.193, df=9	Control vs. CAS	***	*P* < 0.0001
	Descriptive	Control: 10 ± 1.054 (n = 10 fish)			
		CAS: 3.4 ± 0.6532 (n = 10 fish)			
	paired *t*-test (Two-tailed)	t=0, df=9	Control vs. Water	ns	*P* > 0.9999
	Descriptive	Control: 10 ± 1.054 (n = 10 fish)			
		Water: 10 ± 1.054 (n = 10 fish)			
**Fig 1f sound stimu.**	paired *t*-test (Two-tailed)	t=11.07, df=5	Control vs. 5	***	*P* = 0.0001
	Descriptive	Control: 74 ± 1.095 (n = 6 fish)			
		5: 67 ± 1 (n = 6 fish)			
	paired *t*-test (Two-tailed)	t=5.855, df=5	Control vs. 10	**	*P* = 0.0021
	Descriptive	Control: 74 ± 1.095 (n = 6 fish)			

		10: 70 ± 1.571 (n = 6 fish)			
	paired *t*-test (Two-tailed)	t=5.394, df=5	Control vs. 15	**	*P* = 0.0030
	Descriptive	Control: 74 ± 1.095 (n = 6 fish)			
		15: 71.33 ± 1.358 (n = 6 fish)			
	paired *t*-test (Two-tailed)	t=2.236, df=5	Control vs. 20	*	*P* = 0.0356
	Descriptive	Control: 74 ± 1.095 (n = 6 fish)			
		20: 72 ± 1.528 (n = 6 fish)			
	paired *t*-test (Two-tailed)	t=1.581, df=5	Control vs. 25	ns	*P* = 0.1747
	Descriptive	Control: 74 ± 1.095 (n = 6 fish)			
		25: 73.67 ± 1.116 (n = 6 fish)			
	paired *t*-test (Two-tailed)	t=1, df=5	Control vs. 30	ns	*P* = 0.3632
	Descriptive	Control: 74 ± 1.095 (n = 6 fish)			
		30: 74.50 ± 1.285 (n = 6 fish)			
**Fig 1f Elect. stimu.**	paired *t*-test (Two-tailed)	t=19, df=5	Control vs. 5	***	*P* < 0.0001
	Descriptive	Control: 0.45 ± 0.02236 (n = 6 fish)			
		5: 0.1333 ± 0.02108 (n = 6 fish)			
	paired *t*-test (Two-tailed)	t=11.62, df=5	Control vs. 10	***	*P* < 0.0001
	Descriptive	Control: 0.45 ± 0.02236 (n = 6 fish)			
		10: 015. ± 0.02236 (n = 6 fish)			
	paired *t*-test (Two-tailed)	t=11, df=5	Control vs. 15	**	*P* = 0.0011
	Descriptive	Control: 0.45 ± 0.02236 (n = 6 fish)			
		15: 0.2667 ± 0.0333 (n = 6 fish)			
	paired *t*-test (Two-tailed)	t=6.708, df=5	Control vs. 20	**	*P* = 0.0031
	Descriptive	Control: 0.45 ± 0.02236 (n = 6 fish)			
		20: 0.3 ± 0.04472 (n = 6 fish)			
	paired *t*-test (Two-tailed)	t=1.581, df=5	Control vs. 25	ns	*P* = 0.1747
	Descriptive	Control: 0.45 ± 0.02236 (n = 6 fish)			
		25: 0.4167 ± 0.04014 (n = 6 fish)			
	paired *t*-test (Two-tailed)	t=0, df=5	Control vs. 30	ns	*P* > 0.9999
	Descriptive	Control: 0.45 ± 0.02236 (n = 6 fish)			
		30: 0.45 ± 0.02236 (n = 6 fish)			

**Fig 1f HCL stimu.**	paired *t*-test (Two-tailed)	t=10.38, df=5	Control vs. 5	***	*P* = 0.0001
	Descriptive	Control: 0.3 ± 0.2582 (n = 6 fish)			
		5: 0.09167 ± 0.008333 (n = 6 fish)			
	paired *t*-test (Two-tailed)	t=23, df=5	Control vs. 10	***	*P* = 0.0001
	Descriptive	Control: 0.3 ± 0.2582 (n = 6 fish)			
		10: 0.1083 ± 0.02007 (n = 6 fish)			
	paired *t*-test (Two-tailed)	t=6.708, df=5	Control vs. 15	**	*P* = 0.0011
	Descriptive	Control: 0.3 ± 0.2582 (n = 6 fish)			
		15: 0.15 ± 0.02236 (n = 6 fish)			
	paired *t*-test (Two-tailed)	t=3.796, df=5	Control vs. 20	**	*P* = 0.0027
	Descriptive	Control: 0.3 ± 0.2582 (n = 6 fish)			
		20: 0.1833 ± 0.0166 (n = 6 fish)			
	paired *t*-test (Two-tailed)	t=1, df=5	Control vs. 25	ns	*P* = 0.3632
	Descriptive	Control: 0.3 ± 0.2582 (n = 6 fish)			
		25: 0.2833 ± 0.03073 (n = 6 fish)			
	paired *t*-test (Two-tailed)	t=0, df=5	Control vs. 30	ns	*P* > 0.9999
	Descriptive	Control: 0.3 ± 0.2582 (n = 6 fish)			
		30: 0.3 ± 0.2582 (n = 6 fish)			
**Fig 1f Cadav. stimu.**	paired *t*-test (Two-tailed)	t=7.27, df=5	Control vs. 5	***	*P* = 0.0008
	Descriptive	Control: 10.83 ± 1.537 (n = 6 fish)			
		5: 3.0 ± 0.8944 (n = 6 fish)			
	paired *t*-test (Two-tailed)	t=6.299, df=5	Control vs. 10	***	*P* = 0.0009
	Descriptive	Control: 10.83 ± 1.537 (n = 6 fish)			
		10: 0.125 ± 0.8433 (n = 6 fish)			
	paired *t*-test (Two-tailed)	t=5.814, df=5	Control vs. 15	**	*P* = 0.0021
	Descriptive	Control: 10.83 ± 1.537 (n = 6 fish)			
		15: 4.33 ± 0.6667 (n = 6 fish)			
	paired *t*-test (Two-tailed)	t=3.713, df=5	Control vs. 20	**	*P* = 0.0057
	Descriptive	Control: 10.83 ± 1.537 (n = 6 fish)			

		20: 6 ± 1.414 (n = 6 fish)				
	paired *t*-test (Two-tailed)	t=1.581, df=5		Control vs. 25	ns	*P* = 0.1747
	Descriptive	Control: 10.83 ± 1.537 (n = 6 fish)				
		25: 9.167 ± 0.8333 (n = 6 fish)				
	paired *t*-test (Two-tailed)	t=0, df=5		Control vs. 30	ns	*P* > 0.9999
	Descriptive	Control: 10.83 ± 1.537 (n = 6 fish)				
		30: 10.83 ± 1.537 (n = 6 fish)				
**Fig 2g Neuro.Synch.Core.**	paired *t*-test (Two-tailed)	t=23.28, df=6		Control vs. CAS	***	*P* < 0.0001
	Descriptive	Control: 0.002207 ± 0.0006752 (n = 7 fish)				
		CAS: 0.7760 ± 0.03328 (n = 7 fish)				
**Fig 2i Decre.thres.durat.**	One-way ANOVA	F=1.039, P = 0.4034	Tukey's test	sound vs. synch.	ns	*Padj* = 0.7826
				Elect. vs. synch.	ns	*Padj* = 0.5481
				HCL vs. synch.	ns	*Padj* = 0.7826
				Cadav. vs. synch.	ns	*Padj* = 0.3245
	Descriptive	sound :21.43 ± 1.429 (n = 7 fish)				
		Elect. :20.71 ± 1.304 (n = 7 fish)				
		HCL :21.43 ± 0.9221 (n = 7 fish)				
		Cadav. :20 ± 1.091 (n = 7 fish)				
		synch. :23.51 ± 1.573 (n = 7 fish)				
**Fig 2h Frequency**	paired *t*-test (Two-tailed)	t=20.96, df=19		Control vs. CAS	***	*P* < 0.0001
	Descriptive		Control	CAS		
		Minimum	0.25	2.967	Control: 0.9492 ± 0.1173 (n = 20 fish)	
		25% Percentile	0.5333	4.75		
		Median	0.8083	6.05	CAS: 5.844 ± 0.3171 (n = 20 fish)	
		75% Percentile	1.425	7.025		
		Maximum	1.867	7.883		
**Fig 3i Kaede red neurons**	paired *t*-test (Two-tailed)	t=51.35, df=5		0h vs. 6h	***	*P* < 0.0001
	Descriptive	0h:0 ± 0 (n = 6 fish)				

		6h: 82.07 ± 1.592 (n = 6 fish)			
**Fig 3s Neuro.Synch.Core.**	paired *t*-test (Two-tailed)	t=49.33, df=5	*tph2*$^{+/+}$: Control vs. CAS	***	*P* < 0.0001
	Descriptive	Control: 0.002510 ± 0.001582 (n = 6 fish)			
		CAS: 0.7782 ±0.01641 (n = 6 fish)			
	paired *t*-test (Two-tailed)	t=1.103, df=5	*tph2*$^{-/-}$: Control vs. CAS	ns	*P* = 0.3203
	Descriptive	Control: 0.005867 ± 0.005231 (n = 6 fish)			
		CAS: 0.001427 ± 0.001236 (n = 6 fish)			
	paired *t*-test (Two-tailed)	t=20.33, df=5	*tph2*$^{-/-}$: Control vs. 5-HT	***	*P* < 0.0001
	Descriptive	Control: 0.0001142 ± 0.0003186 (n = 6 fish)			
		5-HT: 0.8269 ± 0.04068 (n = 6 fish)			
**Fig 4e Number**	paired *t*-test (Two-tailed)		Osc. vs. Reb.	ns	*P* > 0.9999
	Descriptive	Osc.:7.667 ± 0.6146 (n = 6 fish)			
		Reb.:7.667 ± 0.6146 (n = 6 fish)			
**Fig 4f Number**	paired *t*-test (Two-tailed)	t=11.93, df=5	Osc. vs. Reb	***	*P* < 0.0001
	Descriptive	Osc.:7.167 ± 0.6009 (n = 6 fish)			
		Reb.:0 ± 0 (n = 6 fish)			
**Fig 4j Burst frequency**	paired *t*-test (Two-tailed)	t=10.10, df=7	*htr7a*$^{+/+}$vglut2a:DsRed: Ctrl vs. CAS	***	*P* < 0.0001
	Descriptive	Ctrl: 0.01250 ± 0.005223 (n = 8 fish)			
		CAS: 0.3875 ± 0.03723 (n = 8 fish)			
	paired *t*-test (Two-tailed)	t=1.426, df=7	*htr7a*$^{+/+}$vglut2a:DsRed with SB269970*: Ctrl vs. CAS*	ns	*P* = 0.1970
	Descriptive	Ctrl: 0.02083 ± 0.004167 (n = 8 fish)			
		CAS: 0.02708 ± 0.006250 (n = 8 fish)			
	paired *t*-test (Two-tailed)	t=1, df=7	*htr7a*$^{-/-}$*vglut2a:DsRed: Ctrl vs. CAS*	ns	*P* = 0.3506
	Descriptive	Ctrl: 0.008334 ± 0.003150 (n = 8 fish)			
		CAS: 0.01250 ± 0.004167 (n = 8 fish)			
**Fig 4k Burst frequency**	paired *t*-test (Two-tailed)	t=1, df=7	*htr7a*$^{+/+}$*gad1b:EGFP*: Ctrl vs. CAS	ns	*P* = 0.3506
	Descriptive	Ctrl: 0.006250 ± 0.003050 (n = 8 fish)			
		CAS: 0.004167 ± 0.002728 (n = 8 fish)			

**Fig 4o Neuro.Synch.Core.**	paired *t*-test (Two-tailed)	t=18.54, df=5	*htr7a*$^{+/+}$: Ctrl vs. CAS	***	*P* < 0.0001
	Descriptive	Ctrl: 0.001985 ± 0.0006934 (n = 6 fish)			
		CAS: 0.7881 ± 0.04229 (n = 6 fish)			
	paired *t*-test (Two-tailed)	t=0.4007, df=5	*htr7a*$^{+/+}$ with SB269970: Ctrl vs. CAS	ns	*P* = 0.7052
	Descriptive	Ctrl: 0.001433 ± 0.0001913 (n = 6 fish)			
		CAS: 0.001294 ± 0.0001966 (n = 6 fish)			
	paired *t*-test (Two-tailed)	t=1.530, df=5	*htr7a*$^{-/-}$: Ctrl vs. CAS	ns	*P* = 0.1866
	Descriptive	Ctrl: 0.001773 ± 0.0002334 (n = 6 fish)			
		CAS: 0.001305 ± 0.0002874 (n = 6 fish)			
**Fig 5b Firing frequency**	paired *t*-test (Two-tailed)	t=10.71, df=5	Control *vs.* Opto.	***	*P* = 0.0001
	Descriptive	Control : 1.211 ± 0.07080 (n = 6 fish)			
		Opto.: 7.228 ± 0.5234 (n = 6 fish)			
**Fig 5d Firing frequency**	paired *t*-test (Two-tailed)	t=12.32, df=7	Control *vs.* Opto.	ns	*P* < 0.0001
	Descriptive	Control : 0.01667 ± 0.00498 (n = 8 fish)			
		Opto.: 0.4354 ± 0.03399 (n = 8 fish)			
**Fig 5i sound stimu.**	paired *t*-test (Two-tailed)	t=11.76, df=9	Control vs. ChR2	***	*P* < 0.0001
	Descriptive	Control: 73.7 ± 0.7753 (n = 10 fish)			
		ChR2: 68.1 ± 0.5859 (n = 10 fish)			
	paired *t*-test (Two-tailed)	t=0, df=9	Control vs. mCherry	ns	*P* > 0.9999
	Descriptive	Control: 73.10 ± 0.7667 (n = 10 fish)			
		mCherry: 73.10 ± 0.7667 (n = 10 fish)			
**Fig 5i Elect. stimu.**	paired *t*-test (Two-tailed)	t=8.835, df=9	Control vs. ChR2	***	*P* < 0.0001
	Descriptive	Control: 0.42 ± 0.02906 (n = 10 fish)			
		ChR2: 0.19 ± 0.02769 (n = 10 fish)			
	paired *t*-test (Two-tailed)	t=0, df=9	Control vs. mCherry	ns	*P* > 0.9999
	Descriptive	Control: 0.4 ± 0.02981 (n = 10 fish)			
		mCherry: 0.4 ± 0.02981(n = 10 fish)			
**Fig 5i HCL stimu.**	paired *t*-test (Two-tailed)	t=14.21, df=9	Control vs. ChR2	***	*P* < 0.0001
	Descriptive	Control: 0.3 ± 0.02582 (n = 10 fish)			
		ChR2: 0.1150 ± 0.01979 (n = 10 fish)			

	paired *t*-test (Two-tailed)	t=0, df=9	Control vs. mCherry	ns	*P* > 0.9999
	Descriptive	Control: 0.3 ± 0.02582 (n = 10 fish)			
		mCherry: 0.3 ± 0.02582 (n = 10 fish)			
**Fig 5i Cadav. stimu.**	paired *t*-test (Two-tailed)	t=8.193, df=9	Control vs. ChR2	***	*P* < 0.0001
	Descriptive	Control: 10 ± 1.054 (n = 10 fish)			
		ChR2: 3.4 ± 0.6532 (n = 10 fish)			
	paired *t*-test (Two-tailed)	t=0, df=9	Control vs. mCherry	ns	*P* > 0.9999
	Descriptive	Control: 10.5 ± 0.8975 (n = 10 fish)			
		mCherry: 10.5 ± 0.8975 (n = 10 fish)			
**Fig 5j sound stimu.**	paired *t*-test (Two-tailed)	t=10.5, df=9	Control vs. 5	***	*P* < 0.0001
	Descriptive	Control: 73.7 ± 0.7753 (n = 10 fish)			
		5: 67.2 ± 0.8794 (n = 10 fish)			
	paired *t*-test (Two-tailed)	t=10.17, df=9	Control vs. 10	**	*P* = 0.0014
	Descriptive	Control: 73.7 ± 0.7753 (n = 10 fish)			
		10: 69.4 ± 0.7630 (n = 10 fish)			
	paired *t*-test (Two-tailed)	t=5.842, df=9	Control vs. 15	**	*P* = 0.0022
	Descriptive	Control: 73.7 ± 0.7753 (n = 10 fish)			
		15: 70 ± 0.8692 (n = 10 fish)			
	paired *t*-test (Two-tailed)	t=6.249, df=9	Control vs. 20	**	*P* = 0.0076
	Descriptive	Control: 73.7 ± 0.7753 (n = 10 fish)			
		20: 70.50 ± 0.9458 (n = 10 fish)			
	paired *t*-test (Two-tailed)	t=3.545, df=9	Control vs. 25	*	*P* = 0.0347
	Descriptive	Control: 73.7 ± 0.7753 (n = 10 fish)			
		25: 72.4 ± 0.8192 (n = 10 fish)			
	paired *t*-test (Two-tailed)	t=1, df=9	Control vs. 30	ns	*P* = 0.3434
	Descriptive	Control: 73.7 ± 0.7753 (n = 10 fish)			
		30: 73.6 ± 0.7630 (n = 10 fish)			
**Fig 5j Elect. stimu.**	paired *t*-test (Two-tailed)	t=10.85, df=9	Control vs. 5	***	*P* < 0.0001
	Descriptive	Control: 0.42 ± 0.02906 (n = 10 fish)			
		5: 0.18 ± 0.02494 (n = 10 fish)			

	paired *t*-test (Two-tailed)	t=10.78, df=9	Control vs. 10	**	*P* = 0.0011
	Descriptive	Control: 0.42 ± 0.02906 (n = 10 fish)			
		10: 019. ± 0.02333 (n = 10 fish)			
	paired *t*-test (Two-tailed)	t=7.965, df=9	Control vs. 15	**	*P* = 0.0018
	Descriptive	Control: 0.42 ± 0.02906 (n = 10 fish)			
		15: 0.25 ± 0.03416 (n = 10 fish)			
	paired *t*-test (Two-tailed)	t=6.091, df=9	Control vs. 20	**	*P* = 0.0032
	Descriptive	Control: 0.42 ± 0.02906 (n = 10 fish)			
		20: 0.29 ± 0.03145 (n = 10 fish)			
	paired *t*-test (Two-tailed)	t=2.449, df=9	Control vs. 25	*	*P* = 0.0368
	Descriptive	Control: 0.42 ± 0.02906 (n = 10 fish)			
		25: 0.38 ± 0.02494 (n = 10 fish)			
	paired *t*-test (Two-tailed)	t=0, df=9	Control vs. 30	ns	*P* > 0.9999
	Descriptive	Control: 0.42 ± 0.02906 (n = 10 fish)			
		30: 0.42 ± 0.02906 (n = 10 fish)			
**Fig 5j HCL stimu.**	paired *t*-test (Two-tailed)	t=14.21, df=9	Control vs. 5	***	*P* < 0.0001
	Descriptive	Control: 0.3 ± 0.2582 (n = 10 fish)			
		5: 0.1150 ± 0.01979 (n = 10 fish)			
	paired *t*-test (Two-tailed)	t=12.75, df=9	Control vs. 10	**	*P* = 0.0024
	Descriptive	Control: 0.3 ± 0.2582 (n = 10 fish)			
		10: 0.13 ± 0.02 (n = 10 fish)			
	paired *t*-test (Two-tailed)	t=8.51, df=9	Control vs. 15	**	*P* = 0.0041
	Descriptive	Control: 0.3 ± 0.2582 (n = 10 fish)			
		15: 0.17 ± 0.02134 (n = 10 fish)			
	paired *t*-test (Two-tailed)	t=9, df=9	Control vs. 20	**	*P* = 0.0057
	Descriptive	Control: 0.3 ± 0.2582 (n = 10 fish)			
		20: 0.21 ± 0.02769 (n = 10 fish)			
	paired *t*-test (Two-tailed)	t=2.449, df=9	Control vs. 25	*	*P* = 0.0368
	Descriptive	Control: 0.3 ± 0.2582 (n = 10 fish)			

		25: 0.26 ± 0.01633 (n = 10 fish)				
	paired *t*-test (Two-tailed)	t=0, df=9		Control vs. 30	ns	*P* = 0.3434
	Descriptive	Control: 0.3 ± 0.2582 (n = 10 fish)				
		30: 0.29 ± 0.02333 (n = 10 fish)				
**Fig 5j Cadav. stimu.**	paired *t*-test (Two-tailed)	t=8.573, df=9		Control vs. 5	***	*P* < 0.0001
	Descriptive	Control: 10 ± 1.054 (n = 10 fish)				
		5: 3.0 ± 0.6667 (n = 10 fish)				
	paired *t*-test (Two-tailed)	t=8.193, df=9		Control vs. 10	**	*P* = 0.0019
	Descriptive	Control: 10 ± 1.054 (n = 10 fish)				
		10: 3.4 ± 0.6532 (n = 10 fish)				
	paired *t*-test (Two-tailed)	t=8.034, df=9		Control vs. 15	**	*P* = 0.0032
	Descriptive	Control: 10 ± 1.054 (n = 10 fish)				
		15: 3.8 ± 0.6110 (n = 10 fish)				
	paired *t*-test (Two-tailed)	t=8.820, df=9		Control vs. 20	**	*P* = 0.0074
	Descriptive	Control: 10 ± 1.054 (n = 10 fish)				
		20: 5.6 ± 0.8327 (n = 10 fish)				
	paired *t*-test (Two-tailed)	t=2.979, df=9		Control vs. 25	*	*P* = 0.0155
	Descriptive	Control: 10 ± 1.054 (n = 10 fish)				
		25: 7.6 ± 1.284 (n = 10 fish)				
	paired *t*-test (Two-tailed)	t=1, df=9		Control vs. 30	ns	*P* = 0.3434
	Descriptive	Control: 10 ± 1.054 (n = 10 fish)				
		30: 10.5 ± 1.167 (n = 10 fish)				
**Fig 6c Spike frequency**	One-way ANOVA	F=54.88, P < 0.0001	Tukey's test	Control vs. Persist.	***	*Padj* < 0.0001
				10 Hz vs. Persist.	***	*Padj* < 0.0001
				20 Hz vs. Persist.	***	*Padj* < 0.0001
	Descriptive	Control :0.8967 ± 0.1800 (n = 10 neurons from 3 mice)				
		10 Hz. :1.673 ± 0.2939 (n = 10 neurons from 3 mice)				
		20 Hz :2.793 ± 0.3094 (n = 10 neurons from 3 mice)				
		Persist. :5.83 ± 0.3569 (n = 10 neurons from 3 mice)				

Fig 6j sound stimu. (left)	paired *t*-test (Two-tailed)	t=7.172, df=7	Control vs. hChR2	***	*P* < 0.0001
	Descriptive	Control:79.38 ± 1.475 (n = 8 mice)			
		ChR2: 63.13 ± 1.315 (n = 8 mice)			
	paired *t*-test (Two-tailed)	t=0, df=7	Sb269970: Control vs. hChR2	ns	*P* > 0.9999
	Descriptive	Control: 79.38 ± 1.133 (n = 8 mice)			
		hChR2: 79.38 ± 1.475 (n = 8 mice)			
	paired *t*-test (Two-tailed)	t=0, df=5	Control vs. mCherry	ns	*P* > 0.9999
	Descriptive	Control: 79.17 ± 1.537 (n = 6 mice)			
		mCherry: 79.17 ± 1.537 (n = 6 mice)			
Fig 6j sound stimu. (right)	paired *t*-test (Two-tailed)	t=7.172, df=7	Control vs. 5	***	*P* < 0.0001
	Descriptive	Control: 79.38 ± 1.475 (n = 8 mice)			
		5: 63.13 ± 1.315 (n = 8 mice)			
	paired *t*-test (Two-tailed)	t=9.354, df=7	Control vs. 10	***	*P* < 0.0001
	Descriptive	Control: 79.38 ± 1.475 (n = 8 mice)			
		10: 66.88 ± 0.9149 (n = 8 mice)			
	paired *t*-test (Two-tailed)	t=8.275, df=7	Control vs. 15	***	*P* = 0.0002
	Descriptive	Control: 79.38 ± 1.475 (n = 8 mice)			
		15: 70 ± 0.9449 (n = 8 mice)			
	paired *t*-test (Two-tailed)	t=5, df=7	Control vs. 20	**	*P* = 0.0016
	Descriptive	Control: 79.38 ± 1.475 (n = 8 mice)			
		20: 73.13 ± 0.9149 (n = 8 mice)			
	paired *t*-test (Two-tailed)	t=7, df=7	Control vs. 25	*	*P* = 0.0256
	Descriptive	Control: 79.38 ± 1.475 (n = 8 mice)			
		25: 75 ± 1.336 (n = 8 mice)			
	paired *t*-test (Two-tailed)	t=0, df=7	Control vs. 30	ns	*P* > 0.9999
	Descriptive	Control: 79.38 ± 1.475 (n = 8 mice)			
		30: 79.38 ± 1.475 (n = 8 mice)			
Fig 6k Elect. stimu. (left)	paired *t*-test (Two-tailed)	t=6.614, df=7	Control vs. hChR2	***	*P* = 0.0003
	Descriptive	Control:0.2100 ± 0.006547 (n = 8 mice)			
		ChR2: 0.16 ± 0.005345 (n = 8 mice)			

	paired *t*-test (Two-tailed)	t=0, df=7	Sb269970: Control vs. hChR2	ns	*P* > 0.9999
	Descriptive	Control: 0.2075 ± 0.006478 (n = 8 mice)			
		hChR2: 0.2075 ± 0.005261 (n = 8 mice)			
	paired *t*-test (Two-tailed)	t=0, df=5	Control vs. mCherry	ns	*P* > 0.9999
	Descriptive	Control: 0.21 ± 0.008563 (n = 6 mice)			
		mCherry: 0.21 ± 0.008563 (n = 6 mice)			
**Fig 6j Elect. stimu. (right)**	paired *t*-test (Two-tailed)	t=6.614, df=7	Control vs. 5	***	*P* = 0.0003
	Descriptive	Control: 0.21 ± 0.006547 (n = 8 mice)			
		5: 63.13 ± 1.315 (n = 8 mice)			
	paired *t*-test (Two-tailed)	t=6.333, df=7	Control vs. 10	**	*P* = 0.0024
	Descriptive	Control: 0.21 ± 0.006547 (n = 8 mice)			
		10: 0.1625 ± 0.005901 (n = 8 mice)			
	paired *t*-test (Two-tailed)	t=8.275, df=7	Control vs. 15	**	*P* = 0.0071
	Descriptive	Control: 0.21 ± 0.006547 (n = 8 mice)			
		15: 0.1725 ± 0.005261 (n = 8 mice)			
	paired *t*-test (Two-tailed)	t=5, df=7	Control vs. 20	*	*P* = 0.0223
	Descriptive	Control: 0.21 ± 0.006547 (n = 8 mice)			
		20: 0.1850 ± 0.006268 (n = 8 mice)			
	paired *t*-test (Two-tailed)	t=4.583, df=7	Control vs. 25	*	*P* = 0.0324
	Descriptive	Control: 0.21 ± 0.006547 (n = 8 mice)			
		25: 0.1950 ± 0.005 (n = 8 mice)			
	paired *t*-test (Two-tailed)	t=0, df=7	Control vs. 30	ns	*P* > 0.9999
	Descriptive	Control: 0.21 ± 0.006547 (n = 8 mice)			
		30: 0.21 ± 0.006547 (n = 8 mice)			
**Fig 6l TMT stimu. (left)**	paired *t*-test (Two-tailed)	t=4.583, df=7	Control vs. hChR2	**	*P* = 0.0025
	Descriptive	Control: -3.875 ± 0.2266 (n = 8 mice)			
		ChR2: -4.625 ± 0.2631 (n = 8 mice)			
	paired *t*-test (Two-tailed)	t=0.5517, df=7	Sb269970: Control vs. hChR2	ns	*P* = 0.5983
	Descriptive	Control: -4.125 ± 0.2266 (n = 8 mice)			
		hChR2: -4 ± 0.2673 (n = 8 mice)			

	paired *t*-test (Two-tailed)	t=0, df=5	Control vs. mCherry	ns	*P* > 0.9999
	Descriptive	Control: -4 ± 0.2582 (n = 6 mice)			
		mCherry: -4 ± 0.2582 (n = 6 mice)			
**Fig 6l TMT stimu. (right)**	paired *t*-test (Two-tailed)	t=4.583, df=7	Control vs. 5	**	*P* = 0.0025
	Descriptive	Control: -3.875 ± 0.2266 (n = 8 mice)			
		5: -4.625 ± 0.2631 (n = 8 mice)			
	paired *t*-test (Two-tailed)	t=9, df=7	Control vs. 10	***	*P* < 0.0001
	Descriptive	Control: -3.875 ± 0.2266 (n = 8 mice)			
		10: -5 ± 0.2673 (n = 8 mice)			
	paired *t*-test (Two-tailed)	t=7, df=7	Control vs. 15	**	*P* = 0.0032
	Descriptive	Control: -3.875 ± 0.2266 (n = 8 mice)			
		15: -4.75 ± 0.25 (n = 8 mice)			
	paired *t*-test (Two-tailed)	t=3.416, df=7	Control vs. 20	*	*P* = 0.0112
	Descriptive	Control: -3.875 ± 0.2266 (n = 8 mice)			
		20: -4.5 ± 0.3273 (n = 8 mice)			
	paired *t*-test (Two-tailed)	t=1, df=7	Control vs. 25	ns	*P* = 0.3506
	Descriptive	Control: -3.875 ± 0.2266 (n = 8 mice)			
		25: -4 ± 0.2673 (n = 8 mice)			
	paired *t*-test (Two-tailed)	t=0, df=7	Control vs. 30	ns	*P* > 0.9999
	Descriptive	Control: -3.875 ± 0.2266 (n = 8 mice)			
		30: -3.875 ± 0.2266 (n = 8 mice)			
**Fig 6m 1 st duration (left)**	paired *t*-test (Two-tailed)	t=7.104, df=7	Control vs. hChR2	***	*P* = 0.0002
	Descriptive	Control: 20.63 ± 3.789 (n = 8 mice)			
		ChR2: 76.13 ± 7.913 (n = 8 mice)			
	paired *t*-test (Two-tailed)	t=0.09789, df=7	Sb269970: Control vs. hChR2	ns	*P* = 0.9248
	Descriptive	Control: 20.38 ± 3.959 (n = 8 mice)			
		hChR2: 20.75 ± 3.277 (n = 8 mice)			
	paired *t*-test (Two-tailed)	t=0.3355, df=5	Control vs. mCherry	ns	*P* = 0.7509
	Descriptive	Control: 19.33 ± 4.318 (n = 6 mice)			
		mCherry: 20.5 ± 4.272 (n = 6 mice)			

**Fig 6m Total duration (right)**	paired *t*-test (Two-tailed)	t=9.111, df=7	Control vs. hChR2	***	*P* < 0.0001
	Descriptive	Control: 119.9 ± 16.93 (n = 8 mice)			
		ChR2: 232.5 ± 9.087 (n = 8 mice)			
	paired *t*-test (Two-tailed)	t=1.678, df=7	Sb269970: Control vs. hChR2	ns	*P* = 0.1372
	Descriptive	Control: 121.9 ± 6.786 (n = 8 mice)			
		hChR2: 129.5 ± 5.418 (n = 8 mice)			
	paired *t*-test (Two-tailed)	t=0.2722, df=5	Control vs. mCherry	ns	*P* = 0.7964
	Descriptive	Control: 116.8 ± 17.2 (n = 6 mice)			
		mCherry: 119.5 ± 17.9 (n = 6 mice)			
**Fig 6n 1 st duration (left)**	paired *t*-test (Two-tailed)	t=3.783, df=7	Control vs. hChR2	***	*P* = 0.0009
	Descriptive	Control: 17.75 ± 3.634 (n = 8 mice)			
		ChR2: 31.13 ± 3.847 (n = 8 mice)			
	paired *t*-test (Two-tailed)	t=0.06381, df=7	Sb269970: Control vs. hChR2	ns	*P* = 0.9509
	Descriptive	Control: 17.50 ± 1.604 (n = 8 mice)			
		hChR2: 17.38 ± 1.772 (n = 8 mice)			
	paired *t*-test (Two-tailed)	t=0.2294, df=5	Control vs. mCherry	ns	*P* = 0.8276
	Descriptive	Control: 15.17 ± 2.971 (n = 6 mice)			
		mCherry: 15.50 ± 2.592 (n = 6 mice)			
**Fig 6n Total duration (right)**	paired *t*-test (Two-tailed)	t=6.420, df=7	Control vs. hChR2	***	*P* = 0.0004
	Descriptive	Control: 50.5 ± 4.728 (n = 8 mice)			
		ChR2: 181 ± 22.86 (n = 8 mice)			
	paired *t*-test (Two-tailed)	t=0.2607, df=7	Sb269970: Control vs. hChR2	ns	*P* = 0.8018
	Descriptive	Control: 51.13 ± 2.863 (n = 8 mice)			
		hChR2: 51.88 ± 4.665 (n = 8 mice)			
	paired *t*-test (Two-tailed)	t=0.6384, df=5	Control vs. mCherry	ns	*P* = 0.5513
	Descriptive	Control: 51.83 ± 5.406 (n = 6 mice)			
		mCherry: 54.67 ± 4.349 (n = 6 mice)			
**Fig 6o 1 st duration (left)**	paired *t*-test (Two-tailed)	t=3.349, df=7	Control vs. hChR2	**	*P* = 0.0023
	Descriptive	Control: 21.38 ± 5.144 (n = 8 mice)			
		ChR2: 46.75 ±7.641 (n = 8 mice)			
	paired *t*-test (Two-tailed)	t=0.9721, df=7	Sb269970: Control vs. hChR2	ns	*P* = 0.3634

	Descriptive	Control: 19.13 ± 1.329 (n = 8 mice)				
		hChR2: 20.88 ± 2.371 (n = 8 mice)				
	paired *t*-test (Two-tailed)	t=0.2586, df=5		Control vs. mCherry	ns	*P* = 0.88062
	Descriptive	Control: 19.67 ± 4.349 (n = 6 mice)				
		mCherry: 19.0 ± 2.781 (n = 6 mice)				
**Fig 6o Total duration (right)**	paired *t*-test (Two-tailed)	t=7.842, df=7		Control vs. hChR2	**	*P* = 0.0012
	Descriptive	Control: 96 ± 10.47 (n = 8 mice)				
		ChR2: 230.5 ± 12.03 (n = 8 mice)				
	paired *t*-test (Two-tailed)	t=0.1667, df=7		Sb269970: Control vs. hChR2	ns	*P* = 0.1395
	Descriptive	Control: 90.0 ± 4.814 (n = 8 mice)				
		hChR2: 97.63 ± 4.751 (n = 8 mice)				
	paired *t*-test (Two-tailed)	t=0.1253, df=5		Control vs. mCherry	ns	*P* = 0.9052
	Descriptive	Control: 93.33 ± 6.8 (n = 6 mice)				
		mCherry: 92.17 ± 12.02 (n = 6 mice)				
**Fig 8d Pupil diameter (up)**	One-way ANOVA	F=101, P < 0.0001	Tukey's test	Control vs. Light on	***	*Padj* < 0.0001
				Control vs. Light off	**	*Padj* = 0.0012
				Light on vs. Light off	***	*Padj* < 0.0001
	Descriptive	Control :0.8725 ± 0.05182 (n = 8 mice)				
		Light on :1.783 ± 0.03778 (n = 8 mice)				
		Light off :1.148 ± 0.4858 (n = 8 mice)				
**Fig 8d Pupil diameter (down)**	One-way ANOVA	F=0.047 63, P : ns	Tukey's test	Control vs. Light on	ns	*Padj* =0.9491
				Control vs. Light off	ns	*Padj* = 0.9846
				Light on vs. Light off	ns	*Padj* =0.9892
	Descriptive	Control :0.8738 ± 0.042 (n = 8 mice)				
		Light on :0.86 ± 0.01973 (n = 8 mice)				
		Light off :0.8663 ± 0.02884 (n = 8 mice)				
**Fig 8e Pupil increase duration**	unpaired *t*-test (Two-tailed)	t=39.92, df=14		hChR2 *vs.* mCherry	***	*P* < 0.0001
	Descriptive	hChR2 : 192.5 ± 4.777 (n = 6 fish)				
		mCherry : 1.125 ± 0.3981 (n = 6 fish)				
**Suppl 1b sound stimu. (left)**	paired *t*-test (Two-tailed)	t=10.15, df=9		Control vs. CAS	***	*P* < 0.0001
	Descriptive	Control: 73.5 ± 0.2236 (n = 10 fish)				

		CAS: 68.3 ± 0.5175 (n = 10 fish)			
	paired *t*-test (Two-tailed)	t=0.5571, df=9	Control vs. Water	ns	*P* = 0.5911
	Descriptive	Control: 73.60 ± 0.4761 (n = 10 fish)			
		Water: 73.7 ± 0.4726 (n = 10 fish)			
**Suppl 1b sound stimu. (right)**	paired *t*-test (Two-tailed)	t=10, df=9	Control vs. 5	***	*P* < 0.0001
	Descriptive	Control: 73.5 ± 0.2236 (n = 10 fish)			
		5: 68.3 ± 0.5175 (n = 10 fish)			
	paired *t*-test (Two-tailed)	t=10.8, df=9	Control vs. 10	**	*P* = 0.0032
	Descriptive	Control: 73.5 ± 0.2236 (n = 10 fish)			
		10: 69.3 ± 0.3958 (n = 10 fish)			
	paired *t*-test (Two-tailed)	t=12.75, df=9	Control vs. 15	**	*P* = 0.0041
	Descriptive	Control: 73.5 ± 0.2236 (n = 10 fish)			
		15: 70.1 ± 0.3480 (n = 10 fish)			
	paired *t*-test (Two-tailed)	t=5.811, df=9	Control vs. 20	*	*P* = 0.0125
	Descriptive	Control: 73.5 ± 0.2236 (n = 10 fish)			
		20: 71.2 ± 0.4899 (n = 10 fish)			
	paired *t*-test (Two-tailed)	t=2.377, df=9	Control vs. 25	ns	*P* = 0.1364
	Descriptive	Control: 73.5 ± 0.2236 (n = 10 fish)			
		25: 72.6 ± 0.4522 (n = 10 fish)			
	paired *t*-test (Two-tailed)	t=1, df=9	Control vs. 30	ns	*P* = 0.3434
	Descriptive	Control: 73.5 ± 0.2236 (n = 10 fish)			
		30: 73.6 ± 0.2211 (n = 10 fish)			
**Suppl 1c Elec. stimu. (left)**	paired *t*-test (Two-tailed)	t=0.7571, df=9	Control vs. CAS	***	*P* < 0.0001
	Descriptive	Control: 0.37 ± 0.02134 (n = 10 fish)			
		CAS: 0.15 ± 0.01667 (n = 10 fish)			
	paired *t*-test (Two-tailed)	t=0, df=9	Control vs. Water	ns	*P* > 0.9999
	Descriptive	Control: 0.34 ± 0.03712 (n = 10 fish)			
		Water: 0.34 ± 0.03712 (n = 10 fish)			
**Suppl 1c Elec. stimu. (right)**	paired *t*-test (Two-tailed)	t=7.571, df=9	Control vs. 5	***	*P* < 0.0001
	Descriptive	Control: 0.37 ± 0.02134 (n = 10 fish)			
		5: 0.15 ± 0.01667 (n = 10 fish)			

	paired *t*-test (Two-tailed)	t=9.487, df=9	Control vs. 10	**	*P* = 0.0011
	Descriptive	Control: 0.37 ± 0.02134 (n = 10 fish)			
		10: 017. ± 0.01528 (n = 10 fish)			
	paired *t*-test (Two-tailed)	t=6, df=9	Control vs. 15	**	*P* = 0.0036
	Descriptive	Control: 0.37 ± 0.02134 (n = 10 fish)			
		15: 0.21 ± 0.01 (n = 10 fish)			
	paired *t*-test (Two-tailed)	t=3.857, df=9	Control vs. 20	*	*P* = 0.0213
	Descriptive	Control: 0.37 ± 0.02134 (n = 10 fish)			
		20: 0.28 ± 0.02 (n = 10 fish)			
	paired *t*-test (Two-tailed)	t=1.406, df=9	Control vs. 25	ns	*P* = 0.1747
	Descriptive	Control: 0.37 ± 0.02134 (n = 10 fish)			
		25: 0.34 ± 0.02211 (n = 10 fish)			
	paired *t*-test (Two-tailed)	t=1, df=9	Control vs. 30	ns	*P* = 0.3434
	Descriptive	Control: 0.37 ± 0.02134 (n = 10 fish)			
		30: 0.38 ± 0.02494 (n = 10 fish)			
**Suppl 1d HCL stimu. (left)**	paired *t*-test (Two-tailed)	t=9.391, df=9	Control vs. CAS	***	*P* < 0.0001
	Descriptive	Control: 0.28 ± 0.02494 (n = 10 fish)			
		CAS: 0.1050 ± 0.01740 (n = 10 fish)			
	paired *t*-test (Two-tailed)	t=0, df=9	Control vs. Water	ns	*P* > 0.9999
	Descriptive	Control: 0.27 ± 0.02603 (n = 10 fish)			
		Water: 0.27 ± 0.02603 (n = 10 fish)			
**Suppl 1d HCL stimu. (right)**	paired *t*-test (Two-tailed)	t=9.391, df=9	Control vs. 5	***	*P* < 0.0001
	Descriptive	Control: 0.28 ± 0.02494 (n = 10 fish)			
		5: 0.1050 ± 0.01740 (n = 10 fish)			
	paired *t*-test (Two-tailed)	t=9.391, df=9	Control vs. 10	***	*P* < 0.0001
	Descriptive	Control: 0.28 ± 0.02494 (n = 10 fish)			
		10: 0.1050 ± 0.01740 (n = 10 fish)			
	paired *t*-test (Two-tailed)	t=9, df=9	Control vs. 15	**	*P* = 0.0014
	Descriptive	Control: 0.28 ± 0.02494 (n = 10 fish)			
		15: 0.16 ± 0.02409 (n = 10 fish)			

	paired *t*-test (Two-tailed)	t=6, df=9	Control vs. 20	**	*P* = 0.0136
	Descriptive	Control: 0.28 ± 0.02494 (n = 10 fish)			
		20: 0.21 ± 0.02582 (n = 10 fish)			
	paired *t*-test (Two-tailed)	t=1.964, df=9	Control vs. 25	ns	*P* = 0.0811
	Descriptive	Control: 0.28 ± 0.02494 (n = 10 fish)			
		25: 0.25 ± 0.02236 (n = 10 fish)			
	paired *t*-test (Two-tailed)	t=0, df=9	Control vs. 30	ns	*P* > 0.9999
	Descriptive	Control: 0.28 ± 0.02494 (n = 10 fish)			
		30: 0.28 ± 0.02494 (n = 10 fish)			
**Suppl 1e Cadav. stimu. (left)**	paired *t*-test (Two-tailed)	t=7.672, df=9	Control vs. CAS	***	*P* < 0.0001
	Descriptive	Control: 9.5 ± 1.167 (n = 10 fish)			
		CAS: 3.4 ± 0.6532 (n = 10 fish)			
	paired *t*-test (Two-tailed)	t=0, df=9	Control vs. Water	ns	*P* > 0.9999
	Descriptive	Control: 9.5 ± 1.167 (n = 10 fish)			
		Water: 9.5 ± 1.167 (n = 10 fish)			
**Suppl 1e Cadav. stimu. (right)**	paired *t*-test (Two-tailed)	t=7.27, df=9	Control vs. 5	***	*P* < 0.0001
	Descriptive	Control: 9.5 ± 1.167 (n = 10 fish)			
		5: 3.4 ± 0.6532 (n = 10 fish)			
	paired *t*-test (Two-tailed)	t=7.797, df=9	Control vs. 10	***	*P* < 0.0001
	Descriptive	Control: 9.5 ± 1.167 (n = 10 fish)			
		10: 3.8 ± 0.6110 (n = 10 fish)			
	paired *t*-test (Two-tailed)	t=8.677, df=9	Control vs. 15	**	*P* = 0.0021
	Descriptive	Control: 9.5 ± 1.167 (n = 10 fish)			
		15: 5.2 ± 0.9522 (n = 6 fish)			
	paired *t*-test (Two-tailed)	t=2.979, df=9	Control vs. 20	*	*P* = 0.0155
	Descriptive	Control: 9.5 ± 1.167 (n = 10 fish)			
		20: 7.1 ± 1.038 (n = 10 fish)			
	paired *t*-test (Two-tailed)	t=0, df=9	Control vs. 25	ns	*P* > 0.9999
	Descriptive	Control: 9.5 ± 1.167 (n = 10 fish)			
		25: 9.5 ± 0.8975 (n = 10 fish)			

	paired *t*-test (Two-tailed)	t=1.5, df=9	Control vs. 30	ns	*P* = 0.1679
	Descriptive	Control: 9.5 ± 1.167 (n = 10 fish)			
		30: 10.5 ± 1.167 (n = 10 fish)			
**Suppl 3g Neuro.Synch. Corre.**	paired *t*-test (Two-tailed)	t=9.387, df=4	mCherry : Control vs. CAS	***	*P* = 0.0007
	Descriptive	Control: 0.005187± 0.002971 (n = 5 fish)			
		CAS: 0.7549 ± 0.08098 (n = 5 fish)			
	paired *t*-test (Two-tailed)	t=0.2539, df=4	nfsB-mCherry : Control vs. CAS	ns	*P* = 0.8121
	Descriptive	Control: 0.00083 ± 0.00076 (n = 5 fish)			
		CAS: 0.001185 ± 0.00094 (n = 5 fish)			
**Suppl 4c Burst frequency**	paired *t*-test (Two-tailed)	t=12.37, df=7	*htr7a*$^{+/+}$: Ctrl vs. 5-HT	***	*P* < 0.0001
	Descriptive	Ctrl: 0.01042 ± 0.004384 (n = 8 fish)			
		5-HT: 0.4021 ± 0.02945 (n = 8 fish)			
	paired *t*-test (Two-tailed)	t=2.16, df=7	*htr7a*$^{+/+}$ and SB269970 : Ctrl vs. 5-HT	ns	*P* = 0.0676
	Descriptive	Ctrl: 0.0083 ± 0.004454 (n = 8 fish)			
		5-HT: 0.025 ± 0.008333 (n = 8 fish)			
	paired *t*-test (Two-tailed)	t=0.4236, df=7	*htr7a*$^{-/-}$: Ctrl vs. 5-HT	ns	*P* = 0.6845
	Descriptive	Ctrl: 0.008333 ± 0.004454 (n = 8 fish)			
		5-HT: 0.00625 ± 0.003050 (n = 8 fish)			
**Suppl 4d Neuro.Synch. Corre.**	paired *t*-test (Two-tailed)	t=0.8513, df=4	*htr7a*$^{-/-}$: Ctrl vs. 5-HT	ns	*P* = 0.4426
	Descriptive	Ctrl: 0.001725± 0.0001786 (n = 5 fish)			
		5-HT: 0.001352 ± 0.000386 (n = 5 fish)			
**Suppl 4e Burst frequency**	paired *t*-test (Two-tailed)	t=15.07, df=7	Control : Ctrl vs. CAS	***	*P* < 0.0001
	Descriptive	Ctrl: 0.00833 ± 0.0045 (n = 8 fish)			
		CAS: 0.4354 ± 0.0279 (n = 8 fish)			
	paired *t*-test (Two-tailed)	t=0.314, df=7	NS309 : Ctrl vs. CAS	ns	*P* = 0.7627
	Descriptive	Ctrl: 0.0125 ± 0.005223 (n = 8 fish)			
		CAS: 0.01458 ± 0.004917 (n = 8 fish)			
**Suppl 4f Burst frequency**	paired *t*-test (Two-tailed)	t=11.95, df=7	Control : Ctrl vs. 5-HT	***	*P* < 0.0001
	Descriptive	Ctrl: 0.2083 ± 0.007553 (n = 8 fish)			

		5-HT: 0.4771 ± 0.03377 (n = 8 fish)				
	paired *t*-test (Two-tailed)	t=0.3567, df=7		NS309 : Ctrl vs. 5-HT	ns	*P* = 0.7318
	Descriptive	Ctrl: 0.01667 ± 0.006299 (n = 8 fish)				
		5-HT: 0.1875 ± 0.004917 (n = 8 fish)				
**Suppl 5 Vigilance duration**	One-way ANOVA	F=150, P < 0.0001	Tukey's test	30s-100% vs. 120s-50%	***	*Padj* < 0.0001
				30s-100% vs. 120s-100%	***	*Padj* < 0.0001
				120s-50% vs. 120s-100%	***	*Padj* < 0.0001
	Descriptive	30s-100% :0.85 ± 0.152 (n = 10 mice)				
		120s-50% :2.083 ± 0.2112 (n = 10 mice)				
		120s-100% :5 ± 0.1511 (n = 10 mice)				
**Suppl 6d Spike frequency**	paired *t*-test (Two-tailed)	t=6575, df=6		Control *vs.* mCherry	ns	*P* = 0.5399
	Descriptive	Control : 1.02 ± 0.1559 (n = 6 neurons from 3 mice)				
		mCherry : 0.9783 ± 0.1031 (n = 6 neurons from 3 mice)				
**Suppl 6f Vigilance duration**	One-way ANOVA	F=48.08, P < 0.0001	Tukey's test	Control vs. Light on	***	*Padj* < 0.0001
				Control vs. Light off	*	*Padj* = 0.042
				Light on vs. Light off	***	*Padj* < 0.0001
	Descriptive	Control :15.52 ± 0.9441 (n = 8 mice)				
		Light on :4.896 ± 0.6385 (n = 8 mice)				
		Light off :12.6 ± 0.7624 (n = 8 mice)				
**Suppl 6f Vigilance duration**	One-way ANOVA	F=85.62, P < 0.0001	Tukey's test	Control vs. Light on	***	*Padj* < 0.0001
				Control vs. Light off	***	*Padj* < 0.0001
				Light on vs. Light off	***	*Padj* < 0.0001
	Descriptive	Control :15.63 ± 0.625 (n = 8 mice)				
		Light on :4.167 ± 0.3858 (n = 8 mice)				
		Light off :9.375 ± 0.7835 (n = 8 mice)				
**Suppl 7d Spike frequency**	paired *t*-test (Two-tailed)	t=8.625, df=5		hM3D(Gq) : Control vs. CNO	***	*P* = 0.0003
	Descriptive	Control: 0.8028 ± 0.07846 (n = 6 mice)				
		CNO: 0.47 ± 0.4547 (n = 6 mice)				
	paired *t*-test (Two-tailed)	t=0.1.274, df=5		mCherry : Control vs. CNO	ns	*P* = 0.2586
	Descriptive	Control: 0.8056 ± 0.07505 (n = 6 mice)				

		CNO: 0.7535 ± 0.08236 (n = 6 mice)			
**Suppl 7e Total locomotor distance**	unpaired *t*-test (Two-tailed)	t=12.4, df=10	Control *vs.* CNO	***	*P* < 0.0001
	Descriptive	Control : 17160 ± 528.3 (n = 6 mice)			
		CNO : 7706 ± 549.4 (n = 6 mice)			
**Suppl 7f Total locomotor distance**	unpaired *t*-test (Two-tailed)	t=1.263, df=10	Control *vs.* CNO	ns	*P* = 0.2352
	Descriptive	Control : 17090 ± 1795 (n = 6 mice)			
		CNO : 14690 ± 624.6 (n = 6 mice)			
**Suppl 7g sound stimu. (left)**	paired *t*-test (Two-tailed)	t=9.220, df=5	hM3d(Gq) :Control vs. CNO	***	*P* = 0.0003
	Descriptive	Control:80 ± 1.291 (n = 6 mice)			
		CNO: 65.83 ± 2.007 (n = 6 mice)			
	paired *t*-test (Two-tailed)	t=0, df=5	mCherry: Control vs. CNO	ns	*P* > 0.9999
	Descriptive	Control: 80 ± 1.826 (n = 6 mice)			
		CNO : 80 ± 1.291 (n = 6 mice)			
**Suppl 7g sound stimu. (right)**	paired *t*-test (Two-tailed)	t=9.220, df=5	Control vs. 5	***	*P* = 0.0003
	Descriptive	Control:80 ± 1.291 (n = 6 mice)			
		5: 65.83 ± 2.007 (n = 6 mice)			
	paired *t*-test (Two-tailed)	t=9.220, df=5	Control vs. 10	***	*P* = 0.0003
	Descriptive	Control:80 ± 1.291 (n = 6 mice)			
		10: 65.83 ± 2.007 (n = 6 mice)			
	paired *t*-test (Two-tailed)	t=8.0, df=5	Control vs. 15	***	*P* = 0.0005
	Descriptive	Control:80 ± 1.291 (n = 6 mice)			
		15: 66.67 ± 1.667 (n = 6 mice)			
	paired *t*-test (Two-tailed)	t=5.398, df=5	Control vs. 20	**	*P* = 0.0029
	Descriptive	Control:80 ± 1.291 (n = 6 mice)			
		20: 69.17 ± 2.386 (n = 6 mice)			
	paired *t*-test (Two-tailed)	t=7.746, df=5	Control vs. 25	**	*P* = 0.0056
	Descriptive	Control:80 ± 1.291 (n = 6 mice)			
		25: 70 ±1.826 (n = 6 mice)			
	paired *t*-test (Two-tailed)	t=2.145, df=5	Control vs. 30	*	*P* = 0.0291
	Descriptive	Control:80 ± 1.291 (n = 6 mice)			
		30: 71.67 ± 1.667 (n = 6 mice)			

**Suppl 7h Elect. stimu. (left)**	paired *t*-test (Two-tailed)	t=10.3, df=5	hM3d(Gq) :Control vs. CNO	***	*P* = 0.0001
	Descriptive	Control:0.2233 ± 0.1202 (n = 6 mice)			
		CNO: 0.16 ± 0.007303 (n = 6 mice)			
	paired *t*-test (Two-tailed)	t=1, df=5	mCherry: Control vs. CNO	ns	*P* = 0.3632
	Descriptive	Control: 0.2133 ± 0.01299 (n = 6 mice)			
		CNO: 0.2067 ± 0.006667 (n = 6 mice)			
**Suppl 7h Elect. stimu. (right)**	paired *t*-test (Two-tailed)	t=10.3, df=5	Control vs. 5	***	*P* = 0.0001
	Descriptive	Control:0.2233 ± 0.1202 (n = 6 mice)			
		5: 0.16 ± 0.007303 (n = 6 mice)			
	paired *t*-test (Two-tailed)	t=10.3, df=5	Control vs. 10	***	*P* = 0.0001
	Descriptive	Control:0.2233 ± 0.1202 (n = 6 mice)			
		10: 0.16 ± 0.007303 (n = 6 mice)			
	paired *t*-test (Two-tailed)	t=10.3, df=5	Control vs. 15	***	*P* = 0.0001
	Descriptive	Control:0.2233 ± 0.1202 (n = 6 mice)			
		15: 0.16 ± 0.007303 (n = 6 mice)			
	paired *t*-test (Two-tailed)	t=12.65, df=5	Control vs. 20	**	*P* = 0.0013
	Descriptive	Control:0.2233 ± 0.1202 (n = 6 mice)			
		20: 0.17 ± 0.01 (n = 6 mice)			
	paired *t*-test (Two-tailed)	t=7, df=5	Control vs. 25	**	*P* = 0.0041
	Descriptive	Control:0.2233 ± 0.1202 (n = 6 mice)			
		25: 0.1767 ± 0.009545 (n = 6 mice)			
	paired *t*-test (Two-tailed)	t=7.906, df=5	Control vs. 30	*	*P* = 0.0244
	Descriptive	Control:0.2233 ± 0.1202 (n = 6 mice)			
		30: 0.19 ± 0.01 (n = 6 mice)			
**Suppl 7i TMT stimu. (left)**	paired *t*-test (Two-tailed)	t=7, df=5	hM3d(Gq) :Control vs. CNO	***	*P* = 0.0008
	Descriptive	Control: -3.83 ± 0.3073 (n = 6 mice)			
		CNO: -5 ± 0.3651 (n = 6 mice)			
	paired *t*-test (Two-tailed)	t=0.4152, df=5	mCherry: Control vs. CNO	ns	*P* = 0.6952
	Descriptive	Control: -4 ± 0.2582 (n = 8 mice)			
		CNO: -4.167 ± 0.4014 (n = 8 mice)			
**Suppl 7i TMT stimu. (right)**	paired *t*-test (Two-tailed)	t=7, df=5	Control vs. 5	***	*P* = 0.0008

	Descriptive	Control: -3.83 ± 0.3073 (n = 6 mice)			
		5: -5 ± 0.3651 (n = 6 mice)			
	paired *t*-test (Two-tailed)	t=7, df=5	Control vs. 10	***	*P* = 0.0008
	Descriptive	Control: -3.83 ± 0.3073 (n = 6 mice)			
		10: -5 ± 0.3651 (n = 6 mice)			
	paired *t*-test (Two-tailed)	t=7, df=5	Control vs. 15	***	*P* = 0.0008
	Descriptive	Control: -3.83 ± 0.3073 (n = 6 mice)			
		15: -5 ± 0.3651 (n = 6 mice)			
	paired *t*-test (Two-tailed)	t=4.23, df=5	Control vs. 20	***	*P* = 0.0009
	Descriptive	Control: -3.83 ± 0.3073 (n = 6 mice)			
		20: -4.833 ± 0.3073 (n = 6 mice)			
	paired *t*-test (Two-tailed)	t=5, df=5	Control vs. 25	**	*P* = 0.0041
	Descriptive	Control: -3.83 ± 0.3073 (n = 6 mice)			
		25: -4.667 ± 0.333 (n = 6 mice)			
	paired *t*-test (Two-tailed)	t=2.236, df=5	Control vs. 30	*	*P* = 0.0456
	Descriptive	Control: -3.83 ± 0.3073 (n = 6 mice)			
		30: -4.33 ± 0.2108 (n = 6 mice)			
**Suppl 7j Sound 1 st duration (left)**	paired *t*-test (Two-tailed)	t=17.8, df=5	hM3d(Gq): Control vs. CNO	***	*P* < 0.0001
	Descriptive	Control: 15.83 ± 2.04 (n = 6 mice)			
		CNO: 32.67 ± 1.856 (n = 6 mice)			
	paired *t*-test (Two-tailed)	t=0.7924, df=5	mCherry: Control vs. CNO	ns	*P* = 0.464
	Descriptive	Control: 16.67 ± 2.431 (n = 6 mice)			
		CNO: 18.17 ± 2.798 (n = 6 mice)			
**Suppl 7j Sound Total duration (right)**	paired *t*-test (Two-tailed)	t=8.566, df=5	hM3d(Gq): Control vs. CNO	***	*P* = 0.0004
	Descriptive	Control: 72.17 ± 4.527 (n = 6 mice)			
		CNO: 149.2 ± 6.15 (n = 6 mice)			
	paired *t*-test (Two-tailed)	t=0.9715, df=5	mCherry: Control vs. CNO	ns	*P* = 0.3759
	Descriptive	Control: 71.33 ± 2.261 (n = 6 mice)			
		CNO: 75.00 ± 2.944 (n = 6 mice)			
**Suppl 7k Elect. 1 st duration (left)**	paired *t*-test (Two-tailed)	t=4.695, df=5	hM3d(Gq): Control vs. CNO	**	*P* = 0.0054
	Descriptive	Control: 20.33 ± 3.073 (n = 6 mice)			
		CNO: 32.67 ± 1.856 (n = 6 mice)			

	paired *t*-test (Two-tailed)	t=2.737, df=5		mCherry: Control vs. CNO	ns	*P* = 0.051
	Descriptive	Control: 22.33 ±2.654 (n = 6 mice)				
		CNO: 18.5 ± 1.875 (n = 6 mice)				
**Suppl 7k Elect. Total duration (right)**	paired *t*-test (Two-tailed)	t=6.28, df=5		hM3d(Gq): Control vs. CNO	**	*P* = 0.0015
	Descriptive	Control: 82.17 ± 7.236 (n = 6 mice)				
		CNO: 159.5 ± 8.053 (n = 6 mice)				
	paired *t*-test (Two-tailed)	t=0.4795, df=5		mCherry: Control vs. CNO	ns	*P* = 0.6518
	Descriptive	Control: 79.33 ± 5.725 (n = 6 mice)				
		CNO: 76.83 ± 7.718 (n = 6 mice)				
**Suppl 7l TMT 1 st duration (left)**	paired *t*-test (Two-tailed)	t=9.576, df=5		hM3d(Gq): Control vs. CNO	***	*P* = 0.0002
	Descriptive	Control: 17.17 ± 1.537 (n = 6 mice)				
		CNO: 37.67 ± 2.704 (n = 6 mice)				
	paired *t*-test (Two-tailed)	t=6286, df=5		mCherry: Control vs. CNO	ns	*P* = 0.5572
	Descriptive	Control: 16.5 ± 2.837 (n = 6 mice)				
		CNO: 14.17 ± 2.182 (n = 6 mice)				
**Suppl 7l TMT Total duration (right)**	paired *t*-test (Two-tailed)	t=9.747, df=5		hM3d(Gq): Control vs. CNO	***	*P* = 0.0002
	Descriptive	Control: 64.83 ± 6.69 (n = 6 mice)				
		CNO: 147.7 ± 6.515 (n = 6 mice)				
	paired *t*-test (Two-tailed)	t=0.4682, df=5		mCherry: Control vs. CNO	ns	*P* = 0.6594
	Descriptive	Control: 61.67 ± 9.319 (n = 6 mice)				
		CNO: 64.17 ± 7.153 (n = 6 mice)				
**Suppl 8b Total distance**	One-way ANOVA	F=19.45, P < 0.0001	Tukey's test	Control vs. 5-HT	***	*Padj* < 0.0001
				Control vs. SB269970	ns	*Padj* = 0.4481
				5-HT vs. SB269970	**	*Padj* = 0.0014
	Descriptive	Control :16293 ± 1234 (n = 8 mice)				
		5-HT :7471 ± 688.7 (n = 8 mice)				
		SB269970 :14454 ± 1159 (n = 8 mice)				
**Suppl 8c Sound stimu. (left)**	One-way ANOVA	F=12.95, P = 0.0002	Tukey's test	Control vs. 5-HT	***	*Padj* = 0.0003
				Control vs. SB269970	ns	*Padj* = 0.7821

				5-HT vs. SB269970	**	*Padj* = 0.0017
	Descriptive	Control :79.38 ± 1.133 (n = 8 mice)				
		5-HT :70.63 ± 1.475 (n = 8 mice)				
		SB269970 :78.13 ± 1.315 (n = 8 mice)				
**Suppl 8c sound stimu. (right-5-HT)**	paired *t*-test (Two-tailed)	t=10.69, df=7		Control vs. 5	***	*P* < 0.0001
	Descriptive	Control:79.38 ± 1.133 (n = 8 mice)				
		5: 70.63 ± 1.475 (n = 8 mice)				
	paired *t*-test (Two-tailed)	t=10.69, df=7		Control vs. 10	***	*P* < 0.0001
	Descriptive	Control:79.38 ± 1.133 (n = 8 mice)				
		10: 70.63 ± 1.475 (n = 8 mice)				
	paired *t*-test (Two-tailed)	t=8.881, df=7		Control vs. 15	***	*P* < 0.0001
	Descriptive	Control:79.38 ± 1.133 (n = 8 mice)				
		15: 71.25 ± 1.567 (n = 8 mice)				
	paired *t*-test (Two-tailed)	t=7.937, df=7		Control vs. 20	***	*P* < 0.0001
	Descriptive	Control:79.38 ± 1.133 (n = 8 mice)				
		20: 71.88 ± 1.315 (n = 8 mice)				
	paired *t*-test (Two-tailed)	t=9, df=7		Control vs. 25	**	*P* = 0.0021
	Descriptive	Control:79.38 ± 1.133 (n = 8 mice)				
		25: 73.75 ± 1.25 (n = 8 mice)				
	paired *t*-test (Two-tailed)	t=4.583, df=7		Control vs. 30	ns	*P* = 0.051
	Descriptive	Control:79.38 ± 1.133 (n = 8 mice)				
		30: 77.63 ± 1.475 (n = 8 mice)				
**Suppl 8c sound stimu. (right-SB269970)**	paired *t*-test (Two-tailed)	t=0.7977, df=7		Control vs. 5	ns	*P* = 0.4512
	Descriptive	Control:79.38 ± 1.133 (n = 8 mice)				
		5: 78.13 ± 1.315 (n = 8 mice)				
	paired *t*-test (Two-tailed)	t=0.7977, df=7		Control vs. 10	ns	*P* = 0.4512
	Descriptive	Control:79.38 ± 1.133 (n = 8 mice)				
		10: 78.13 ± 1.315 (n = 8 mice)				
	paired *t*-test (Two-tailed)	t=0.5517, df=7		Control vs. 15	ns	*P* = 0.5983
	Descriptive	Control:79.38 ± 1.133 (n = 8 mice)				
		15: 78.75 ± 1.25 (n = 8 mice)				

	paired *t*-test (Two-tailed)	t=1, df=7		Control vs. 20	ns	*P* = 0.3506
	Descriptive	Control:79.38 ± 1.133 (n = 8 mice)				
		20: 78.75 ± 0.8183 (n = 8 mice)				
	paired *t*-test (Two-tailed)	t=0, df=7		Control vs. 25	ns	*P > 0.9999*
	Descriptive	Control:79.38 ± 1.133 (n = 8 mice)				
		25: 79.38 ± 1.133 (n = 8 mice)				
	paired *t*-test (Two-tailed)	t=1.528, df=7		Control vs. 30	ns	*P* = 0.1705
	Descriptive	Control:79.38 ± 1.133 (n = 8 mice)				
		30: 80.63 ± 1.133 (n = 8 mice)				
**Suppl 8d Elect. stimu. (left)**	One-way ANOVA	F=11.38, P = 0.0004	Tukey's test	Control vs. 5-HT	***	*Padj* = 0.0005
				Control vs. SB269970	ns	*Padj* = 0.4976
				5-HT vs. SB269970	**	*Padj* = 0.0067
	Descriptive	Control : 0.2 ± 0.007559 (n = 8 mice)				
		5-HT : 0.16 ± 0.005345 (n = 8 mice)				
		SB269970 : 0.19 ± 0.005345 (n = 8 mice)				
**Suppl 8d Elect. stimu. (right-5-HT)**	paired *t*-test (Two-tailed)	t=7.483, df=7		Control vs. 5	***	*P* = 0.0001
	Descriptive	Control :0.2 ± 0.007559 (n = 8 mice)				
		5: 0.16 ± 0.005345 (n = 8 mice)				
	paired *t*-test (Two-tailed)	t=7.483, df=7		Control vs. 10	***	*P* = 0.0001
	Descriptive	Control :0.2 ± 0.007559 (n = 8 mice)				
		10: 0.16 ± 0.005345 (n = 8 mice)				
	paired *t*-test (Two-tailed)	t=6.177, df=7		Control vs. 15	***	*P* = 0.0005
	Descriptive	Control :0.2 ± 0.007559 (n = 8 mice)				
		15: 1.675 ± 0.005261 (n = 8 mice)				
	paired *t*-test (Two-tailed)	t=5.227, df=7		Control vs. 20	***	*P* = 0.0002
	Descriptive	Control :0.2 ± 0.007559 (n = 8 mice)				
		20: 0.1725 ± 0.00366 (n = 8 mice)				
	paired *t*-test (Two-tailed)	t=9, df=7		Control vs. 25	**	*P* = 0.0032
	Descriptive	Control :0.2 ± 0.007559 (n = 8 mice)				
		25: 0.1775 ± 0.005901 (n = 8 mice)				

	paired *t*-test (Two-tailed)	t=1.528, df=7		Control vs. 30	ns	*P* = 0.1705
	Descriptive	Control :0.2 ± 0.007559 (n = 8 mice)				
		30: 0.195 ± 0.005 (n = 8 mice)				
**Suppl 8d Elect. stimu. (right-SB269970)**	paired *t*-test (Two-tailed)	t=1.871, df=7		Control vs. 5	ns	*P* = 0.1036
	Descriptive	Control :0.2 ± 0.007559 (n = 8 mice)				
		5: 0.19 ± 0.005345 (n = 8 mice)				
	paired *t*-test (Two-tailed)	t=2.646, df=7		Control vs. 10	*	*P* = 0.0331
	Descriptive	Control :0.2 ± 0.007559 (n = 8 mice)				
		10: 0.19 ± 0.005345 (n = 8 mice)				
	paired *t*-test (Two-tailed)	t=0.5517, df=7		Control vs. 15	ns	*P* = 0.1705
	Descriptive	Control :0.2 ± 0.007559 (n = 8 mice)				
		15: 0.195 ± 0.005 (n = 8 mice)				
	paired *t*-test (Two-tailed)	t=1, df=7		Control vs. 20	ns	*P* = 0.3506
	Descriptive	Control :0.2 ± 0.007559 (n = 8 mice)				
		20: 0.1975 ± 0.005901 (n = 8 mice)				
	paired *t*-test (Two-tailed)	t=0, df=7		Control vs. 25	ns	*P* = 0.3506
	Descriptive	Control :0.2 ± 0.007559 (n = 8 mice)				
		25: 0.2025 ± 0.007008 (n = 8 mice)				
	paired *t*-test (Two-tailed)	t=1, df=7		Control vs. 30	ns	*P* = 0.1705
	Descriptive	Control :0.2 ± 0.007559 (n = 8 mice)				
		30: 0.2 ± 0.007559 (n = 8 mice)				
**Suppl 8e TMT stimu. (left)**	One-way ANOVA	F=5.102, P = 0.0156	Tukey's test	Control vs. 5-HT	*	*Padj* = 0.0479
				Control vs. SB269970	ns	*Padj* = 0.9070
				5-HT vs. SB269970	*	*Padj* = 0.0199
	Descriptive	Control : -3.75 ± 0.25 (n = 8 mice)				
		5-HT : -4.5 ± 0.1890 (n = 8 mice)				
		SB269970 : -3.625 ± 0.1830 (n = 8 mice)				
**Suppl 8e TMT stimu. (right-5-HT)**	paired *t*-test (Two-tailed)	t=2.393, df=7		Control vs. 5	*	*P* = 0.0479
	Descriptive	Control : -3.75 ± 0.25 (n = 8 mice)				
		5: -4.5 ± 0.1890 (n = 8 mice)				

	paired *t*-test (Two-tailed)	t=5.292, df=7	Control vs. 10	**	*P* = 0.0011
	Descriptive	Control : -3.75 ± 0.25 (n = 8 mice)			
		10: -4.75 ± 0.1637 (n = 8 mice)			
	paired *t*-test (Two-tailed)	t=3.862, df=7	Control vs. 15	**	*P* = 0.0062
	Descriptive	Control : -3.75 ± 0.25 (n = 8 mice)			
		15: -4.625 ± 0.183 (n = 8 mice)			
	paired *t*-test (Two-tailed)	t=4.583, df=7	Control vs. 20	**	*P* = 0.0025
	Descriptive	Control : -3.75 ± 0.25 (n = 8 mice)			
		20: -4.5 ± 0.189 (n = 8 mice)			
	paired *t*-test (Two-tailed)	t=2.049, df=7	Control vs. 25	ns	*P* = 0.0796
	Descriptive	Control : -3.75 ± 0.25 (n = 8 mice)			
		25: 4.125 ± 0.2266 (n = 8 mice)			
	paired *t*-test (Two-tailed)	t=1.528, df=7	Control vs. 30	ns	*P* = 0.1705
	Descriptive	Control : -3.75 ± 0.25 (n = 8 mice)			
		30: -4 ± 0.189 (n = 8 mice)			
**Suppl 8e TMT. stimu. (right-SB269970)**	paired *t*-test (Two-tailed)	t=0.5517, df=7	Control vs. 5	ns	*P* = 0.5983
	Descriptive	Control : -3.75 ± 0.25 (n = 8 mice)			
		5: -3.625 ± 0.1830 (n = 8 mice)			
	paired *t*-test (Two-tailed)	t=1, df=7	Control vs. 10	ns	*P* > 0.9999
	Descriptive	Control : -3.75 ± 0.25 (n = 8 mice)			
		10: -3.75 ± 0.25 (n = 8 mice)			
	paired *t*-test (Two-tailed)	t=1, df=7	Control vs. 15	ns	*P* > 0.9999
	Descriptive	Control : -3.75 ± 0.25 (n = 8 mice)			
		15: -3.75 ± 0.25 (n = 8 mice)			
	paired *t*-test (Two-tailed)	t=1, df=7	Control vs. 20	ns	*P* > 0.9999
	Descriptive	Control : -3.75 ± 0.25 (n = 8 mice)			
		20: -3.75 ± 0.25 (n = 8 mice)			
	paired *t*-test (Two-tailed)	t=1, df=7	Control vs. 25	ns	*P* > 0.9999
	Descriptive	Control : -3.75 ± 0.25 (n = 8 mice)			
		25: -3.75 ± 0.25 (n = 8 mice)			
	paired *t*-test (Two-tailed)	t=1, df=7	Control vs. 30	ns	*P* > 0.9999

	Descriptive	Control : -3.75 ± 0.25 (n = 8 mice)				
		30: -3.75 ± 0.25 (n = 8 mice)				
**Suppl 8f Sound 1 st duration (left)**	One-way ANOVA	F=10.96, P = 0.0006	Tukey's test	Control vs. 5-HT	***	*Padj* = 0.0006
				Control vs. SB269970	ns	*Padj* = 0.6058
				5-HT vs. SB269970	**	*Padj* = 0.006
	Descriptive	Control :17 ± 2.726 (n = 8 mice)				
		5-HT :33.13 ± 2.961 (n = 8 mice)				
		SB269970 :20.50 ± 1.871 (n = 8 mice)				
**Suppl 8f Sound Total duration (right)**	One-way ANOVA	F=48.31, P < 0.0001	Tukey's test	Control vs. 5-HT	***	*Padj* < 0.0001
				Control vs. SB269970	ns	*Padj* = 0.1009
				5-HT vs. SB269970	***	*Padj* < 0.0001
	Descriptive	Control :54.5 ± 2.291 (n = 8 mice)				
		5-HT :146.6 ± 10.73 (n = 8 mice)				
		SB269970 :75.75 ± 4.905 (n = 8 mice)				
**Suppl 8g Elect. 1 st duration (left)**	One-way ANOVA	F=25.54, P < 0.0001	Tukey's test	Control vs. 5-HT	***	*Padj* < 0.0001
				Control vs. SB269970	*	*Padj* = 0.0263
				5-HT vs. SB269970	***	*Padj* = 0.0009
	Descriptive	Control :19.38 ± 2.507 (n = 8 mice)				
		5-HT :59.88 ± 5.709 (n = 8 mice)				
		SB269970 :35.5 ± 3.157 (n = 8 mice)				
**Suppl 8g Elect. Total duration (right)**	One-way ANOVA	F=66.08, P < 0.0001	Tukey's test	Control vs. 5-HT	***	*Padj* < 0.0001
				Control vs. SB269970	ns	*Padj* = 0.1628
				5-HT vs. SB269970	***	*Padj* < 0.0001
	Descriptive	Control :76.58 ± 8.111 (n = 8 mice)				
		5-HT :208.1 ± 8.875 (n = 8 mice)				
		SB269970 :99.75 ± 8.916 (n = 8 mice)				
**Suppl 8h TMT 1 st duration (left)**	One-way ANOVA	F=31.72, P < 0.0001	Tukey's test	Control vs. 5-HT	***	*Padj* < 0.0001
				Control vs. SB269970	*	*Padj* = 0.1096

				5-HT vs. SB269970	***	*Padj* < 0.0001
	Descriptive	Control :18.88 ± 2.287 (n = 8 mice)				
		5-HT :42.50 ± 2.478 (n = 8 mice)				
		SB269970 :25.38 ± 1.647 (n = 8 mice)				
**Suppl 8h TMT Total duration (right)**	One-way ANOVA	F=50.59, P < 0.0001	Tukey's test	Control vs. 5-HT	***	*Padj* < 0.0001
				Control vs. SB269970	**	*Padj* = 0.0021
				5-HT vs. SB269970	***	*Padj* < 0.0001
	Descriptive	Control :56.38 ± 4.395 (n = 8 mice)				
		5-HT :147.6 ± 9.492 (n = 8 mice)				
		SB269970 :92.38 ± 3.982 (n = 8 mice)				
**Suppl 9c Pupil diameter**	One-way ANOVA	F=60.54, P < 0.0001	Tukey's test	Control vs. 40 min	***	*Padj* < 0.0001
				Control vs. 120 min	**	*Padj* < 0.0001
				40 min vs. 120 min	*	*Padj* = 0.0108
	Descriptive	Control :0.7913 ± 0.08209 (n = 6 mice)				
		40 min :1.733 ± 0.05721 (n = 6 mice)				
		120 min :1.437 ± 0.03839 (n = 6 mice)				
**Suppl 9e Pupil diameter**	One-way ANOVA	F=0.09374, P = ns	Tukey's test	Control vs. 40 min	ns	*Padj* = 0.9803
				Control vs. 120 min	ns	*Padj* = 0.9683
				40 min vs. 120 min	ns	*Padj* = 0.9029
	Descriptive	Control :0.7913 ± 0.08209 (n = 6 mice)				
		40 min :1.733 ± 0.05721 (n = 6 mice)				
		120 min :1.437 ± 0.03839 (n = 6 mice)				
**Suppl 10d Pupil diameter**	One-way ANOVA	F=50.35, P < 0.0001	Tukey's test	Control vs. Light on	***	*Padj* < 0.0001
				Control vs. Light off	*	*Padj* = 0.0148
				Light on vs. Light off	***	*Padj* < 0.0001
	Descriptive	Control :0.8963 ± 0.04957 (n = 8 mice)				
		Light on :1.544 ± 0.03732 (n = 8 mice)				
		Light off :1.1 ± 0.05179 (n = 8 mice)				

	One-way ANOVA	F=0.09820, P = ns	Tukey's test	Control vs. Light on	ns	*Padj* = 0.9074
				Control vs. Light off	ns	*Padj* = 0.9417
				Light on vs. Light off	ns	*Padj* = 0.9955
	Descriptive	Control :0.8825 ± 0.04431 (n = 8 mice)				
		Light on :0.8650 ± 0.01793 (n = 8 mice)				
		Light off :0.8688 ± 0.01757 (n = 8 mice)				
**Suppl 10e Pupil increase duration**	unpaired *t*-test (Two-tailed)	t=35.26, df=14		hChR2 *vs.* mCherry	***	$P < 0.0001$
	Descriptive	hChR2 : 181.8 ± 5.112 (n = 8 mice)				
		mCherry : 1 ± 0.378 (n = 8 mice)				
**Suppl 11a htr1aa**	unpaired *t*-test (Two-tailed)	t=8.037, df=10		WT *vs. htr1aa*$^{-/-}$	***	$P < 0.0001$
	Descriptive	WT : 1.001 ± 0.0326 (n= 3 technical replicates of 20 pooled fish)				
		htr1aa$^{-/-}$: 0.7124 ± 0.01519 (n= 3 technical replicates of 20 pooled fish)				
**Suppl 11b htr2ab**	unpaired *t*-test (Two-tailed)	t=5.679, df=10		WT *vs. htr2ab*$^{-/-}$	***	$P = 0.0002$
	Descriptive	WT : 1.017 ± 0.08323 (n= 3 technical replicates of 20 pooled fish)				
		htr2ab$^{-/-}$: 0.3737 ± 0.07678 (n= 3 technical replicates of 20 pooled fish)				
**Suppl 11c htr4**	unpaired *t*-test (Two-tailed)	t=10.49, df=10		WT *vs. htr4*$^{-/-}$	***	$P < 0.0001$
	Descriptive	WT : 1.003 ± 0.03314 (n= 3 technical replicates of 20 pooled fish)				
		htr4$^{-/-}$: 0.5898 ± 0.02124 (n= 3 technical replicates of 20 pooled fish)				
**Suppl 11d htr7a**	unpaired *t*-test (Two-tailed)	t=8.868, df=10		WT *vs. htr7a*$^{-/-}$	***	$P < 0.0001$
	Descriptive	WT : 1.002 ± 0.02638 (n= 3 technical replicates of 20 pooled fish)				
		htr7a$^{-/-}$: 0.5256 ± 0.04676 (n= 3 technical replicates of 20 pooled fish)				
