# Supplementary material for: Serotonergic modulation of vigilance states in zebrafish and mice
Source: Nat Commun. 2024 Mar 22;15:2596. doi: 10.1038/s41467-024-47021-0 (PMC10959952; doi:10.1038/s41467-024-47021-0)
Supplement: Supplementary file 3 — Description of Additional Supplementary Information [file 41467_2024_47021_MOESM3_ESM.pdf]

Description of Additional Supplementary Files

File Name : Supplementary Movie 1

Description: CAS induced vigilance behavior in zebrafish compared to that seen with water administration. The timeline is shown on top of the tank in the video with two-minute animal behavior recording before water/CAS injection, water/CAS injection and two-minute recording after water/CAS injection.

File Name : Supplementary Movie 2

Description: The volumetric imaging of the whole dorsal pallium in six layers showing the synchronized neuronal oscillations after CAS application in zebrafish. Neuron positions and calcium oscillation traces are indicated in pink. Recording time 7 min, playing speed 7.5X.

File Name : Supplementary Movie 3

Description: Abolition of CAS-induced vigilance behavior in *htr7a*$^{-/-}$ *vglut2a:DsRed* zebrafish but not in *htr7a*$^{+/+}$ *vglut2a:DsRed* zebrafish. The timeline is shown on top of the tank in the video with two-minute animal behavior recording before CAS injection, CAS injection and two-minute recording after CAS injection.

File Name : Supplementary Movie 4

Description: Optogenetic stimulation of 5-HT neurons generated vigilance behavior in *tph2:ChR2-mCherry* zebrafish compared with *tph2:mCherry* zebrafish. The timeline is shown on top of the tank in the video with two-minute animal behavior recording before light on, two-minute recording with light on and two-minute recording after light on.

File Name : Supplementary Movie 5

Description: Persistent optogenetic stimulation but not pulsed optogenetic stimulation of DRN 5-HT neurons induced locomotor-restricted behavior in pAAV-Ef1a-DIO-hChR2-mCherry transfected Slc6a4-Cre mice. The timeline is shown on top of the video with five-minute animal behavior recording before light on, two-minute recording with pulsed or persistent light stimulation and five-minute recording after light on. Playing speed 3X.
