# Supplementary material for: Serotonergic modulation of vigilance states in zebrafish and mice
Source: Nat Commun. 2024 Mar 22;15:2596. doi: 10.1038/s41467-024-47021-0 (PMC10959952; doi:10.1038/s41467-024-47021-0)
Supplement: Supplementary file 9 — Reporting Summary [file 41467_2024_47021_MOESM9_ESM.pdf]

nature portfolio

Corresponding author(s): Jianren Song

Last updated by author(s): Mar 4, 2024

Reporting Summary

Nature Portfolio wishes to improve the reproducibility of the work that we publish. This form provides structure for consistency and transparency in reporting. For further information on Nature Portfolio policies, see our Editorial Policies and the Editorial Policy Checklist.

Statistics

For all statistical analyses, confirm that the following items are present in the figure legend, table legend, main text, or Methods section.

n/a	Confirmed	
☐	☒	The exact sample size (*n*) for each experimental group/condition, given as a discrete number and unit of measurement
☐	☒	A statement on whether measurements were taken from distinct samples or whether the same sample was measured repeatedly
☐	☒	The statistical test(s) used AND whether they are one- or two-sided<br>*Only common tests should be described solely by name; describe more complex techniques in the Methods section.*
☒	☐	A description of all covariates tested
☐	☒	A description of any assumptions or corrections, such as tests of normality and adjustment for multiple comparisons
☐	☒	A full description of the statistical parameters including central tendency (e.g. means) or other basic estimates (e.g. regression coefficient) AND variation (e.g. standard deviation) or associated estimates of uncertainty (e.g. confidence intervals)
☐	☒	For null hypothesis testing, the test statistic (e.g. *F*, *t*, *r*) with confidence intervals, effect sizes, degrees of freedom and *P* value noted<br>*Give P values as exact values whenever suitable.*
☒	☐	For Bayesian analysis, information on the choice of priors and Markov chain Monte Carlo settings
☒	☐	For hierarchical and complex designs, identification of the appropriate level for tests and full reporting of outcomes
☐	☒	Estimates of effect sizes (e.g. Cohen's *d*, Pearson's *r*), indicating how they were calculated

Our web collection on statistics for biologists contains articles on many of the points above.

Software and code

Policy information about availability of computer code

Data collection	Clampex v10.7.0.3, SciScan 1.3, CoreView software v2.1.0.33,ZebraZoom v1.17,Smart V3.0,Oculomatic Pro 1.9.7
Data analysis	Prism 9.0 Fiji v1.53c, Matlab R2018a v9.4, Clampfit v10.6,Photoshop CC 2018,Premiere Pro CC 2018,NeuroExplorer 5,Spike2 7.2,edgeR (v 3.14.0),Offline Sorter x64 V4

For manuscripts utilizing custom algorithms or software that are central to the research but not yet described in published literature, software must be made available to editors and reviewers. We strongly encourage code deposition in a community repository (e.g. GitHub). See the Nature Portfolio guidelines for submitting code & software for further information.

Data

Policy information about availability of data

All manuscripts must include a data availability statement. This statement should provide the following information, where applicable:

- Accession codes, unique identifiers, or web links for publicly available datasets
- A description of any restrictions on data availability
- For clinical datasets or third party data, please ensure that the statement adheres to our policy

Raw and processed RNA-seq data generated in this study are deposited into the GEO database with accession number GSE253039. Source data are provided with this paper.

Research involving human participants, their data, or biological material

Policy information about studies with human participants or human data. See also policy information about sex, gender (identity/presentation), and sexual orientation and race, ethnicity and racism.

Reporting on sex and gender	N/A
Reporting on race, ethnicity, or other socially relevant groupings	N/A
Population characteristics	N/A
Recruitment	N/A
Ethics oversight	N/A

Note that full information on the approval of the study protocol must also be provided in the manuscript.

Field-specific reporting

Please select the one below that is the best fit for your research. If you are not sure, read the appropriate sections before making your selection.

☒ Life sciences ☐ Behavioural & social sciences ☐ Ecological, evolutionary & environmental sciences

For a reference copy of the document with all sections, see nature.com/documents/nr-reporting-summary-flat.pdf

Life sciences study design

All studies must disclose on these points even when the disclosure is negative.

Sample size	Sample-size is estimated base on 3R principles, how large a difference, how much variability,"p" value and the confidence intervals. We used online spreadsheet (Lamorte's Power Calculations) to estimate the sample size.
Data exclusions	No data were excluded from the analyses.
Replication	At least three measures were taken to verify the reproducibility of the experimental findings. All attempts at replication were successful.
Randomization	All samples were randomly allocated into experimental groups.
Blinding	The investigators were blinded to group allocation during data collection and analysis.

Reporting for specific materials, systems and methods

We require information from authors about some types of materials, experimental systems and methods used in many studies. Here, indicate whether each material, system or method listed is relevant to your study. If you are not sure if a list item applies to your research, read the appropriate section before selecting a response.

Materials & experimental systems

n/a	Involved in the study	
☐	☒	Antibodies
☒	☐	Eukaryotic cell lines
☒	☐	Palaeontology and archaeology
☐	☒	Animals and other organisms
☒	☐	Clinical data
☒	☐	Dual use research of concern
☒	☐	Plants

Methods

n/a	Involved in the study	
☒	☐	ChIP-seq
☒	☐	Flow cytometry
☒	☐	MRI-based neuroimaging

Antibodies

Antibodies used	1. Rabbit Anti-Serotonin Antibody, Unconjugated, Supplier Name: Sigma-Aldrich, Cat#: S5545, lot#: 087M4758V<br>2. Alexa Fluor 488-AffiniPure Donkey Anti-Rabbit IgG (H+L) antibody, Supplier Name: Jackson ImmunoResearch Labs, Cat#: 711-545-152, lot#: 144917

3.mCherry Monoclonal Antibody (16D7),Supplier Name: Thermo Fisher,Cat#: M11217, lot#: XJ359389
4.Alexa Fluor 568 Goat anti-Rat IgG (H+L) antibody, Supplier Name: Thermo Fisher, Cat#: A11077, lot#: 2026150

Validation

1. Rabbit Anti-Serotonin Antibody (Cat#: S5545); species: human, rat; application: immunohistochemistry (formalin-fixed, paraffin-embedded sections), immunohistochemistry (frozen sections); manufacturer's website: https://www.sigmaaldrich.com/catalog/product/sigma/s5545?lang=zh&region=CN
2.mCherry Monoclonal Antibody (16D7)(Cat#: M11217);species: Tag; application:immunohistochemistry (formalin-fixed, paraffin-embedded sections), immunohistochemistry (frozen sections); manufacturer's website: https://www.thermofisher.cn/cn/zh/antibody/product/mCherry-Antibody-clone-16D7-Monoclonal/M11217

Animals and other research organisms

Policy information about studies involving animals; ARRIVE guidelines recommended for reporting animal research, and Sex and Gender in Research

Laboratory animals

All experiments were performed using 4-5 weeks old fish with mixed gender and male Slc6a4-Cre mice of two-month old.
1. Wild-type zebrafish AB line;
2. Tg(tph2:GFP)
3. Tg(tph2:Gal4)
4. Tg(UAS:GCaMP6)
5. Tg(UAS:nfsB-mcherry)
6. Tg(vglut2a:loxP-DsRed-loxP-Gal4)
7. Tg(HuC:H2B-GCaMP6f)
8. TgBAC(gad1b:EGFP)
9.Tg(tph2:Kaede)
10.Tg(5×UAS:ChR2(H134R)-mCherry)
11.Tg(UAS:mCherry)
12. Slc6a4-Cre mice

Wild animals

The study did not involve wild animals.

Reporting on sex

In this study, mixed gender zebrafish and male mice were used for all experiments. Sex-based analyses were not performed since the reporting effects and findings were not supposed to be sex relevant.

Field-collected samples

The study did not involve samples collected from the field.

Ethics oversight

All the experimental protocols were approved by the Animal Use Committee of Tongji University.

Note that full information on the approval of the study protocol must also be provided in the manuscript.

Plants

Seed stocks

Report on the source of all seed stocks or other plant material used. If applicable, state the seed stock centre and catalogue number. If plant specimens were collected from the field, describe the collection location, date and sampling procedures.

Novel plant genotypes

Describe the methods by which all novel plant genotypes were produced. This includes those generated by transgenic approaches, gene editing, chemical/radiation-based mutagenesis and hybridization. For transgenic lines, describe the transformation method, the number of independent lines analyzed and the generation upon which experiments were performed. For gene-edited lines, describe the editor used, the endogenous sequence targeted for editing, the targeting guide RNA sequence (if applicable) and how the editor was applied.

Authentication

Describe any authentication procedures for each seed stock used or novel genotype generated. Describe any experiments used to assess the effect of a mutation and, where applicable, how potential secondary effects (e.g. second site T-DNA insertions, mosiacism, off-target gene editing) were examined.
